# Auxin-driven patterning with unidirectional fluxes

**DOI:** 10.1093/jxb/erv262

**Published:** 2015-06-27

**Authors:** Mikolaj Cieslak, Adam Runions, Przemyslaw Prusinkiewicz

**Affiliations:** Department of Computer Science, University of Calgary, 2500 University Dr. N.W., Calgary, AB T2N 1N4, Canada

**Keywords:** Petri nets, auxin-driven patterning, canalization, convergence point formation, dual polarization, modulated feedback, polar auxin transport, unidirectional flux.

## Abstract

The plant hormone auxin plays an essential role in the patterning of plant structures. Biological hypotheses supported by computational models suggest that auxin may fulfil this role by regulating its own transport, but the plausibility of previously proposed models has been questioned. We applied the notion of unidirectional fluxes and the formalism of Petri nets to show that the key modes of auxin-driven patterning—the formation of convergence points and the formation of canals—can be implemented by biochemically plausible networks, with the fluxes measured by dedicated tally molecules or by efflux and influx carriers themselves. Common elements of these networks include a positive feedback of auxin efflux on the allocation of membrane-bound auxin efflux carriers (PIN proteins), and a modulation of this allocation by auxin in the extracellular space. Auxin concentration in the extracellular space is the only information exchanged by the cells. Canalization patterns are produced when auxin efflux and influx act antagonistically: an increase in auxin influx or concentration in the extracellular space decreases the abundance of efflux carriers in the adjacent segment of the membrane. In contrast, convergence points emerge in networks in which auxin efflux and influx act synergistically. A change in a single reaction rate may result in a dynamic switch between these modes, suggesting plausible molecular implementations of coordinated patterning of organ initials and vascular strands predicted by the dual polarization theory.

## Highlight

The key modes of auxin-driven patterning— the formation of convergence points and canals—depend on whether auxin efflux and influx feed back on polar auxin transport synergistically or antagonistically.

## Introduction

The plant hormone auxin plays an essential role regulating key aspects of plant development. At least some of the multiple functions of auxin, such as patterning of vascular strands, positioning of organ primordia, and regulation of leaf form, are attributed to the self-organization of auxin transport: a feedback process in which auxin affects the distribution of its transporters (Berleth *et al.*, 2007; Scarpella *et al.*, 2010). The nature of this feedback has been the focal point of many studies, reviewed recently by Habets and Offringa (2014). They include the construction of computational models, which aim at identifying interactions that could lead to the observed spatio-temporal patterns of auxin activity and localization of auxin transporters (reviewed by Prusinkiewicz and Runions, 2012; van Berkel *et al.*, 2013; Runions *et al.*, 2014).

Many experimental data support auxin canalization, the concept proposed by Sachs (1969, 1991) to explain the vascular patterning in plants, and first modelled by Mitchison (1980, 1981). The canalization hypothesis postulates a positive feedback between net auxin flux and the localization of auxin transporters (‘with-the-flux polarization’). However, as pointed out by multiple authors (Merks *et al.*, 2007; Kramer, 2009; Wabnik *et al.*, 2010; Abley *et al.*, 2013; Bennett *et al.*, 2014), how this feedback can be implemented in nature has remained unclear. According to the law of mass action, the rates of chemical reactions depend on the concentrations of reactants rather than their fluxes.

A different hypothesis, which postulates the localization of auxin efflux carriers towards cells with high auxin concentration (‘up-the-gradient’ model; Jönsson *et al.*, 2006; Smith *et al.*, 2006), was proposed to explain the emergence of auxin maxima involved in the patterning of organ primordia (Reinhardt *et al.*, 2003) and serration and lobes in leaves (Hay *et al.*, 2006; Scarpella *et al.*, 2006). In this case, it is not clear through which locally operating mechanism cells could respond to the auxin concentration in their neighbours (Kramer, 2009; Wabnik *et al.*, 2010; Abley *et al.*, 2013; Bennett *et al.*, 2014). Furthermore, the assumption that auxin efflux carriers are polarized by two qualitatively different mechanisms has been questioned (Merks *et al.*, 2007; Stoma *et al.*, 2008).

Coen *et al.* (2004) hypothesized that the measurement of auxin fluxes can be effected by tally molecules, produced or consumed in auxin transport processes. The term ‘measurement’ denotes here a process in which the concentration of some substance becomes proportional to the flux, or, more generally, is a function of the flux. We modify and extend this concept by considering unidirectional fluxes [see Ussing (1949) for the original exploration, Appendix 4 in Chen and Eisenberg (1993) for general definitions, and Eisenberg (2012, p. 166) for a survey of the relevant literature]. In contrast to net fluxes, which characterize the difference in the number of molecules crossing a surface in opposite directions, a unidirectional flux characterizes the flow of a substance in one direction; the counterflow is neglected. We observe that a cell can plausibly use common biochemical reactions, such as transport with Michaelis–Menten kinetics (Edelstein-Keshet, 2005), to measure unidirectional fluxes. Furthermore, the measurement of unidirectional fluxes in opposite directions provides more information than the measurement of the net flux. Using an analogy with road traffic, net flux does not allow us to distinguish between a country road with no cars and a divided highway with an equally high car flow in both directions: the measurement of two independent variables, such as the unidirectional flow in each direction, is needed to distinguish these cases. A similar observation applies to the auxin flux: no movement of auxin molecules across a membrane and high efflux matched by an equally high influx both yield a net flux of zero. A distinction between these cases provides additional information that a cell may employ for polarization and patterning purposes. We consider the information provided by separate measurements of auxin efflux and influx through the cell membrane, and show that networks in which these measurements feed back on auxin transport yield biochemically plausible implementations of basic auxin-driven patterning modes. These modes are commonly classified as with-the-flux, up-the-gradient, and dual polarization models (Bayer *et al.*, 2009; Runions *et al.*, 2014).

We begin by characterizing basic functional modules (which we refer to as motifs) that a cell may use to measure auxin efflux or influx through a membrane. We then show how these motifs can be integrated into simple reaction networks (referred to as circuits) that provide information about the difference of influx and outflux (i.e. the net flux), their ratio, or other combinations of influx and outflux. Finally, we examine several models in which this information feeds back on the allocation of auxin transporters to membranes. Using simulations of two-dimensional tissues, we show that the proposed models can exhibit both with-the-flux and up-the-gradient behaviour. Furthermore, we note that the transition between both types of behaviour, postulated by the dual polarization model (Bayer *et al.*, 2009), does not require a change in network configuration, and in some networks can be effected by only changing one reaction rate.

## Methods

### Modelling reaction and transport networks

We apply the notion of Petri nets (reviewed, for example, by Murata, 1989; Heiner *et al.*, 2008; Blätke *et al.*, 2011) to define and compare the biochemical networks involved in auxin transport and patterning. The graphical character of Petri nets facilitates the intuitive understanding and comparisons of the networks and processes under consideration.

**Fig. 1.**
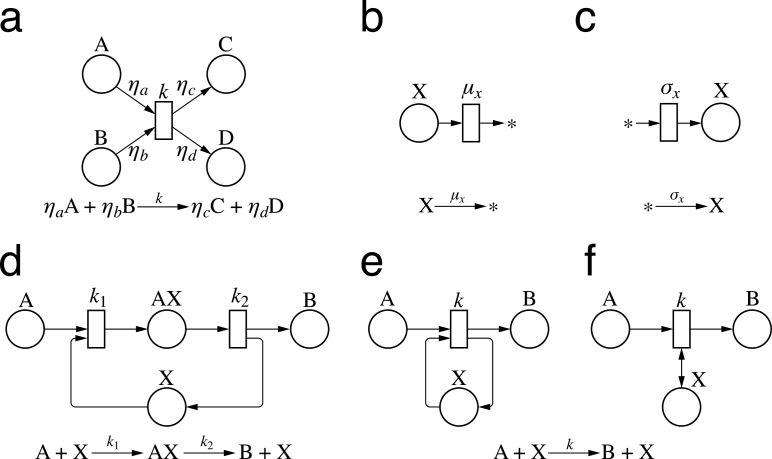
Examples of Petri nets representing chemical reactions. (a) A generalized synthesis/decomposition reaction. Numbers associated with arcs indicate stoichiometric coefficients. (b) Turnover of molecule X into a product outside the scope of interest. (c) Production of molecule X from a substrate outside the scope of interest. (d–f) Three representations of a catalytic reaction. The intermediate product AX is shown explicitly in case (d) but omitted in cases (e) and (f). The separate arcs representing consumption and reproduction of catalyst X (e) are collapsed into a bidirectional arrow in (f).

A Petri net is a directed graph with two types of nodes: *places*, drawn as circles, and *transitions*, drawn as rectangles (Fig. 1a). When applied to describe biochemical processes, places represent molecular species and transitions represent reactions. The nodes are connected by directed arcs (arrows), such that the arcs pointing from places to transitions denote the reactants, and arcs pointing from transitions to places denote the products. Arcs connecting places to places or transitions to transitions are not allowed. If a reaction involves two or more molecules of the same species, the number of molecules is noted as a label associated with the arc.

The conventions used in this paper are shown in Fig. 1b–f. We use the symbol * as a general descriptor of reactants and products outside the modelled system (c.f. Gillespie, 1976). For example, Fig. 1b represents turnover of substance X, where the reaction product leaves the system under consideration or is not of interest. Likewise, Fig. 1c represents the production of X, where the reactants summarized by symbol * are abundant and can support the production of X at a constant rate $\left(\sigma_{x}\right)$ without being depleted. Intermediate products of catalytic reactions, shown explicitly in Fig. 1d (AX), are sometimes omitted to make presentation more compact (Fig. 1e, f). In the latter case, arcs indicating consumption and recreation of the catalyst are depicted individually (Fig. 1e) or collapsed into a single bidirectional arrow (Fig. 1f).

According to their original definition, Petri nets are a discrete formalism: in biochemical applications, they operate on integer numbers of molecules, and individual reactions are fired at discrete time points. Discrete Petri nets are directly applicable to the stochastic modelling and simulation of processes in which the numbers of molecules are small (Goss and Peccoud, 1998). It is likely, however, that the numbers of molecules involved in auxin transport and auxin-driven patterning are sufficiently large to be treated as continuous variables. Consequently, in this paper, we use the continuous extension of Petri nets (Heiner *et al.*, 2008). This means, in particular, that the labels associated with transitions indicate reaction rate constants rather than reaction propensities.

Consistent with the assumption of continuity, we model reaction kinetics using systems of ordinary differential equations (Edelstein-Keshet, 2005). For simplicity, we assume that all reactions are elementary, and thus stoichiometric coefficients determine orders of reactions with respect to the reactants. In the mathematical analysis of reaction networks, we assume quasi-steady conditions. We note that the equations representing the quasi-steady state can be written directly by inspecting Petri nets and applying the laws of mass action and mass conservation. For example, the steady-state concentration of the intermediate product AX in Fig. 1d satisfies the equation:

$$k_{1}[\text{A}][\text{X}]=k_{2}[\text{AX}]$$ (1)

**Fig. 2.**
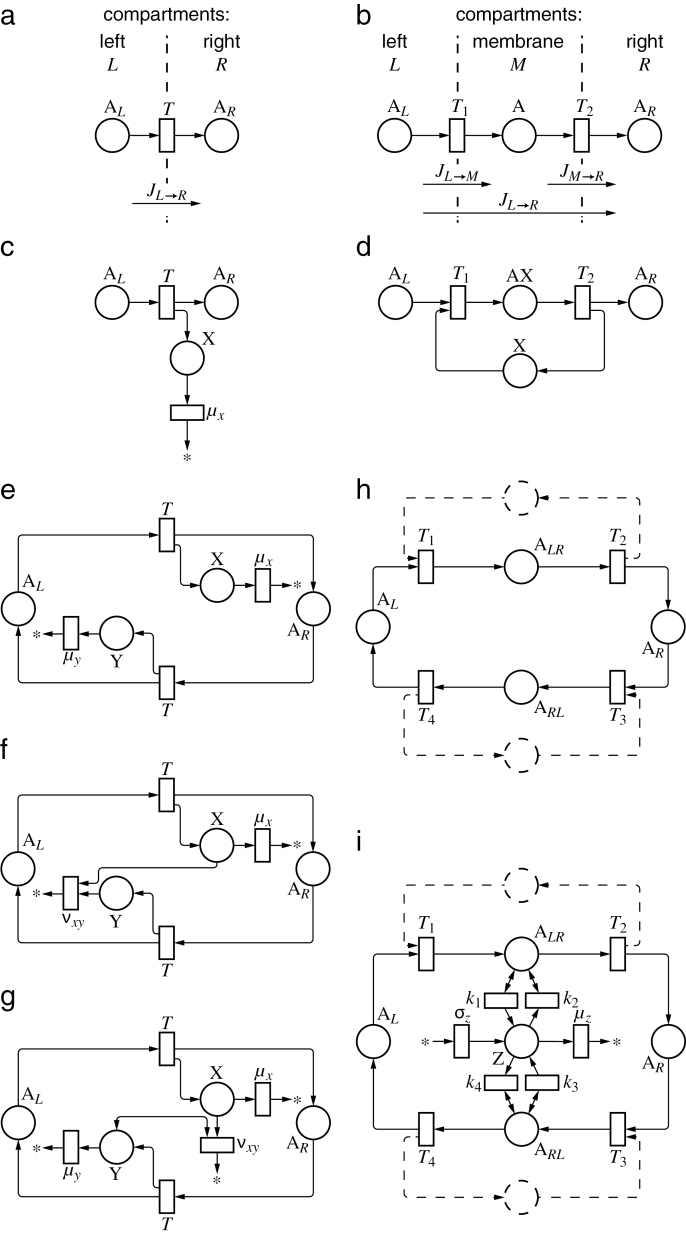
Petri net representation of transport processes and circuits measuring unidirectional fluxes. (a) Unidirectional flux $J_{L\to R}$ from compartment *L* to compartment *R* is proportional to the concentration of the transported substance, [A_*L*_]. (b) Unidirectional flux through a membrane compartment *M* is proportional to the steady-state concentration of the transported substance in the membrane, [A]. (c) Unidirectional flux of A from compartment *L* to compartment *R* is measured by the concentration of tally molecule, [X]. (d) Unidirectional flux from *L* to *R* is measured by the concentration of transporter X bound to the transported molecule A, [AX]. (e) A pair of processes (c) is combined to measure unidirectional fluxes in both directions. (f) Concentration of X measures net flux from *L* to *R*. (g) Concentration of X measures the ratio of unidirectional fluxes. (h) A pair of processes (b) or (d) is combined to measure unidirectional fluxes in both directions. Dashed lines indicate a possible role of additional molecules, such as molecule X in (d). (i) Concentration of Z measures the ratio of weighted sums of unidirectional fluxes.

The movement of molecules between compartments, such as the cytosol and a cell membrane, is treated as a reaction. For instance, Fig. 2a shows the Petri net describing unidirectional transport between two compartments, *L* and *R*. Fig. 2b extends this example with the inclusion of a membrane *M* between these compartments:

$${\text{A}}_{L}\overset{T_{1}}{\to }\text{A}\overset{T_{2}}{\to }{\text{A}}_{R}$$ (2)

Petri net transitions represent the flow of mass. The volume of compartments must thus be considered when their content is described in terms of concentrations. For example, if Eqn 2 represents transport between compartments of volumes *V^L^*, *V* and *V^R^*, the quasi-steady-state concentrations satisfy the equation:

$$V_{L}T_{1}[{\text{A}}_{L}]=VT_{2}[\text{A}]$$ (3)

Because of the multiplicative character of this relationship, in the remainder of this paper we assume that the impact of compartment volumes *V* is subsumed in reaction constants *T*.

We further assume that interfaces between adjacent cells have unit area. This assumption simplifies presentation by equating mass flow with fluxes, and can be removed using standard techniques for modelling arbitrary cellular networks (for example, see Jönsson *et al.*, 2006; Smith *et al.*, 2006).

### Implementation and visualization of simulations

**Fig. 3.**
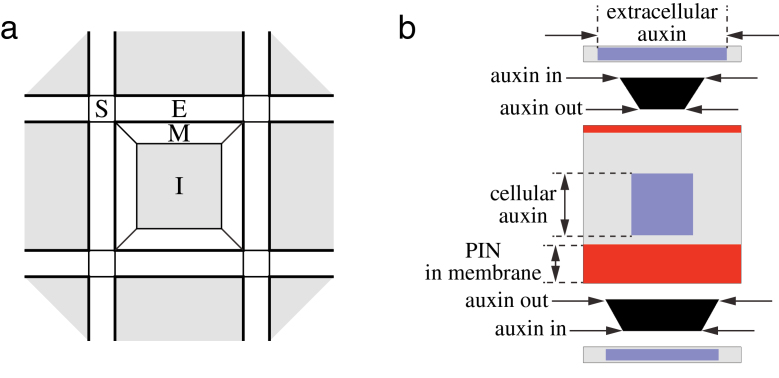
(a) Definition of the cell arrangement used in simulations. Each cell consists of the interior compartment I and four membrane compartments M. Extracellular space is a network of compartments E that separate adjacent cells and are connected by small compartments S at the cell corners. (b) Visualization of cells, extracellular space, and fluxes used in the simulations. The size of squares and rectangles indicates the concentration of auxin and PIN. Cells and intercellular compartments are graphically separated, providing room for a representation of fluxes (black trapezoids) between cell membranes and extracellular compartments. The length of the edges ‘auxin in’ and ‘auxin out’ indicates unidirectional auxin fluxes entering and leaving the cell (influx and efflux through membrane segments). (This figure is available in colour at *JXB* online.)

Given Petri net models of biochemical networks operating at the level of individual cells, we model the emerging patterns by assembling the cells into a square grid (Fig. 3a). Each cell comprises five compartments: the cell interior (cytosol) and four membrane sections facing the adjacent cells. Except for the first simulation, which explores Mitchison’s (1980, 1981) canalization model that ignored extracellular space, we assume that cells are separated by extracellular space. This space is modelled as a network of interconnected compartments. The rectangularly shaped compartments separating adjacent cells are connected by square compartments at the junctions of four cells (Fig. 3a). Auxin can accumulate in all extracellular compartments and flow through them. We assume that various chemical species are well mixed, so that their concentrations are uniform within each intra- or extracellular compartment. The concentration of auxin in the extracellular space is the only information that adjacent cells share.

For simulation purposes, we modelled the reaction and transport kinetics using systems of ordinary differential equations based directly on Petri nets and extended from individual cells to cell assemblies. We solved these equations numerically using the implicit backward differentiation formula (BDF) method implemented in the C-language package CVODE from the SUite of Nonlinear and DIfferential/ALgebraic equation Solvers (SUNDIALS) (Hindmarsh *et al.*, 2005). The values of parameters used in all simulations are given in Supplementary Tables S1 and S2, available at *JXB* online. They are reported to assure reproducibility of our results, rather than to provide the physical values. Given the hypothetical character of the models, experimental data on which parameters could be based are not yet available. All simulation models have been specified in the L+C programming language (Karwowski and Prusinkiewicz, 2003; Prusinkiewicz *et al.*, 2007), and executed using the *lpfg* simulator (Karwowski and Prusinkiewicz, 2003) included in the *Virtual Laboratory* package (Federl and Prusinkiewicz, 1999). The simulations are visualized using the graphical convention explained in Fig. 3b.

## Results

### Unidirectional fluxes can be measured by concentrations

The notion that fluxes affect biochemical processes may appear at odds with the law of mass action, according to which the rate of a chemical reaction depends on the concentrations of reactants rather than their fluxes. We show that this is not a real antinomy, because unidirectional fluxes can be measured indirectly, by the concentration of the transported substance, the concentration of transporters bound to the transported substance, or the concentration of substances produced or consumed during transport events.

#### Concentration of transported substance

In the case of diffusive transport of a substance A between compartments *L* and *R* (Fig. 2a), or active transport when transporters are abundant, the unidirectional flux $J_{L\to R}$ from *L* to *R* is proportional to the concentration [A_*L*_] of A in the source compartment *L*. The equation:

$$J_{L\to R}=T[{\text{A}}_{L}]$$ (4)

which follows from the law of mass action, thus makes it possible to determine the flux of A, given its concentration.

#### Concentration of tally molecules

Unidirectional fluxes can also be measured when transport events are accompanied by the production or consumption of some molecule X. Following Coen *et al.* (2004), we refer to such molecules as tally molecules. The Petri net in Fig. 2c defines a process in which tally molecules are produced by transport events and turned over at a constant rate. In the steady state, the production and turnover balance each other:

$$J_{L\to R}-\mu_{x}[\text{X}]=0$$ (5)

or

$$[\text{X}]=\frac{J_{L\to R}}{\mu_{x}}$$ (6)

The concentration [X] is thus a measure of the unidirectional flux of A from *L* to *R*.

#### Concentration of transporters

Fig. 2d can be interpreted as a sample Petri net representing active transport from compartment *L* to compartment *R* via membrane *M*. The transporter X may be free or bound to the transported molecule, forming complex AX. According to the law of mass action, the flux $J_{M\to R}$ of A from the membrane to the compartment *R* is proportional to the concentration [AX] of the bound transporter: $J_{M\to R}=k_{2}[\text{AX}]$ . If the system is in a (quasi)steady state, the concentration [AX] is approximately constant. The influx of A to compartment *M* is then equal to its efflux from compartment *M* to *R* and to the overall steady-state unidirectional flux of A from *L* to *R*:

$$J_{L\to M}=J_{M\to R}=J_{L\to R}=T_{2}[\text{AX}]$$ (7)

where the notation for fluxes is as in Fig. 2b. The concentration [AX] of bound transporters is thus proportional to, and hence a measure of, the unidirectional transport of A from *L* to *R*:

$$[\text{AX}]=\frac{J_{L\to R}}{T_{2}}$$ (8)

The above equation also holds if the reaction binding transporter X to A is reversible. The transport process then has Michaelis–Menten kinetics, an exemplar for both enzymatic reactions and active transport (Edelstein-Keshet, 2005), which further confirms that unidirectional fluxes can be measured by concentrations of substances involved in common reactions.

### Functions of unidirectional fluxes can be computed by plausible biochemical circuits

It is conceivable that unidirectional fluxes feed back on auxin transport indirectly, through biochemical circuits that compute functions of fluxes. Examples of such circuits are given below.

### Net flux

The circuit in Fig. 2e combines two motifs from Fig. 2c, and thus measures unidirectional fluxes of substance A in both directions. A simple modification, in which molecules X and Y can annihilate each other, but Y alone is not subject to turnover, results in a circuit that measures (positive) net flux from left to right (Fig. 2f). Specifically, in a steady state we have:

$$J_{L\to R}-\mu_{x}[X]-\nu_{xy}[X][Y]=0$$ (9)

$$J_{R\to L}-\nu_{xy}\left[X\right]\left[Y\right]=0$$ (10)

By subtracting these equations, we obtain:

$$J_{L\to R}-J_{R\to L}-\mu_{x}[X]=0$$ (11)

or

$$[\text{X}]=\frac{\phi_{L\to R}}{\mu_{x}}$$ (12)

where $\phi_{L\to R}=J_{L\to R}-J_{R\to L}$ is the net flux from *L* to *R*. We notice that, for $\phi_{L\to R}<0$ , Eqn 12 formally yields negative concentrations [X]. Since concentrations cannot be negative, we need to consider this case separately. From Fig. 2f, it is evident that, for $J_{L\to R}<J_{R\to L}$ , the flux from *L* to *R* is too small to prevent unbounded growth of concentration [Y]. The system is thus not in a steady state, and Eqns 9–12 do not apply. If $\nu_{xy}>0$ , the increasing abundance of molecules Y brings the concentration [X] asymptotically to zero. By combining Eqn 12 with this observation, we obtain:

$$[\text{X}]=\{\begin{array}{ll} \frac{1}{\mu_{x}}\phi_{L\to R} & \text{if }\phi_{L\to R}\geq 0\\ 0 & \text{if }\phi_{L\to R}<0 \end{array}$$ (13)

#### Flux ratio

A different modification of the circuit in Fig. 2e yields the circuit in Fig. 2g, which computes the ratio of fluxes rather than the net flux. In this case, Y catalyses the turnover of X. In a steady state, the system satisfies equations:

$$J_{L\to R}-\mu_{x}[X]-\nu_{xy}[X][Y]=0$$ (14)

$$J_{R\to L}-\mu_{y}[Y]=0$$ (15)

By solving these equations for [X], we obtain:

$$[X]=\frac{\mu_{y}J_{L\to R}}{\nu_{xy}J_{R\to L}+\mu_{x}\mu_{y}}$$ (16)

#### Ratio of weighted sums of fluxes

Functions of fluxes can also be computed on the basis of the motif of Fig. 2d. The circuit of Fig. 2h combines two such motifs, and thus measures unidirectional fluxes of substance A in both directions. Fig. 2i presents a further extension, in which both intermediate complexes can catalyse production or turnover of an additional molecule Z. In the steady state, the concentration of molecule Z satisfies the equation:

$$\begin{array}{l} k_{1}[A_{LR}]-k_{2}[A_{LR}][Z]+k_{3}[A_{RL}]-\\ k_{4}[A_{RL}][Z]+\sigma_{z}-\mu_{z}[Z]=0 \end{array}$$ (17)

and thus:

$$[\text{Z}]=\frac{k_{1}[A_{LR}]+k_{3}[A_{RL}]+\sigma_{z}}{k_{2}[A_{LR}]+k_{4}[A_{RL}]+\mu_{z}}$$ (18)

The steady-state concentrations of the molecules A_*LR*_ and A_*RL*_ are proportional to the unilateral fluxes: $[A_{LR}]=J_{L\to R}/T_{2}$ and $[{\text{A}}_{RL}]=J_{R\to L}/T_{4}$ (see Section ‘Concentration of transporters’). By substituting these fluxes for concentrations in Eqn 18, we obtain:

$$[Z]=\frac{\kappa_{1}J_{L\to R}+\kappa_{3}J_{R\to L}+\sigma_{z}}{\kappa_{2}J_{L\to R}+\kappa_{4}J_{R\to L}+\mu_{z}}$$ (19)

where $\kappa_{1}=k_{1}/T_{2}$ , $\kappa_{2}=k_{2}/T_{2}$ , $\kappa_{3}=k_{3}/T_{4}$ , and $\kappa_{4}=k_{4}/T_{4}$ . The circuit of Fig. 2i thus computes the ratio of weighted sums of fluxes. Depending on the values of reaction and transport constants, the fluxes $J_{L\to R}$ and $J_{R\to L}$ act synergistically to increase the concentration of Z (for instance, if $\kappa_{1}>0,\kappa_{3}>0$ and $\kappa_{2}=\kappa_{4}=0$ ) or act antagonistically (for instance, if $\kappa_{1}>0,\kappa_{4}>0$ and $\kappa_{2}=\kappa_{3}=0$ ). This switch leads to a possible implementation of the dual polarization model (see Section ‘Bound transporters may feed back on PIN localization via an intermediate molecule’).

### Different polarization models can be implemented by plausible networks

Studies of emergent cell polarization have been focused on two fundamental processes: canalization, leading to the patterning of vascular strands, and convergence point formation, leading to the patterning of organ primordia (see ‘Introduction’). Their integration is also of interest, as it appears to be responsible for the redirection of auxin flow at the convergence points: towards a convergence point in the epidermis and away from it in the subepidermal tissues (Bayer *et al.*, 2009; O’Connor *et al.*, 2014). We show that these processes can be implemented in networks that adhere to the basic laws of mass action, and in this sense are biochemically plausible. In this section, we focus on models employing tally molecules.

#### Mitchison’s canalization model

The model of auxin canalization proposed by Mitchison (1980, 1981) and revisited by Rolland-Lagan and Prusinkiewicz (2005) was the first computational model of auxin-based patterning. Its essential element is the regulation of auxin transporters in different segments of the cell membrane according to the net fluxes through these segments. According to the restatement of Mitchison’s equations proposed by Rolland-Lagan and Prusinkiewicz (2005), this regulation is captured by the equations:

$$\frac{dP_{i}}{dt}=\{\begin{array}{ll} \alpha \phi_{i\to j}^{2}+\beta -\gamma P_{i} & \text{if }\phi_{i\to j}\geq 0\\ \beta -\gamma P_{i} & \text{if }\phi_{i\to j}<0 \end{array}$$ (20)

$$\frac{dP_{j}}{dt}=\{\begin{array}{ll} \alpha \phi_{i\to j}^{2}+\beta -\gamma P_{j} & \text{if }\phi_{i\to j}\leq 0\\ \beta -\gamma P_{j} & \text{if }\phi_{i\to j}>0 \end{array}$$ (21)

where *P*_*i*_ and *P*_*j*_ are concentrations of auxin efflux carriers, currently interpreted as the PIN proteins localized to the segment of the membrane of cell *i* abutting cell *j* and to the corresponding segment of the membrane in cell *j* abutting cell *i*, respectively. The parameters α, β, and γ are assumed to be constant. In contrast to Rolland-Lagan and Prusinkiewicz (2005), we do not assume that *P*_*i*_ and *P*_*j*_ are clamped to a maximum *P*_max_, as this assumption is not necessary and has only a small quantitative impact on the simulation results.

**Fig. 4.**
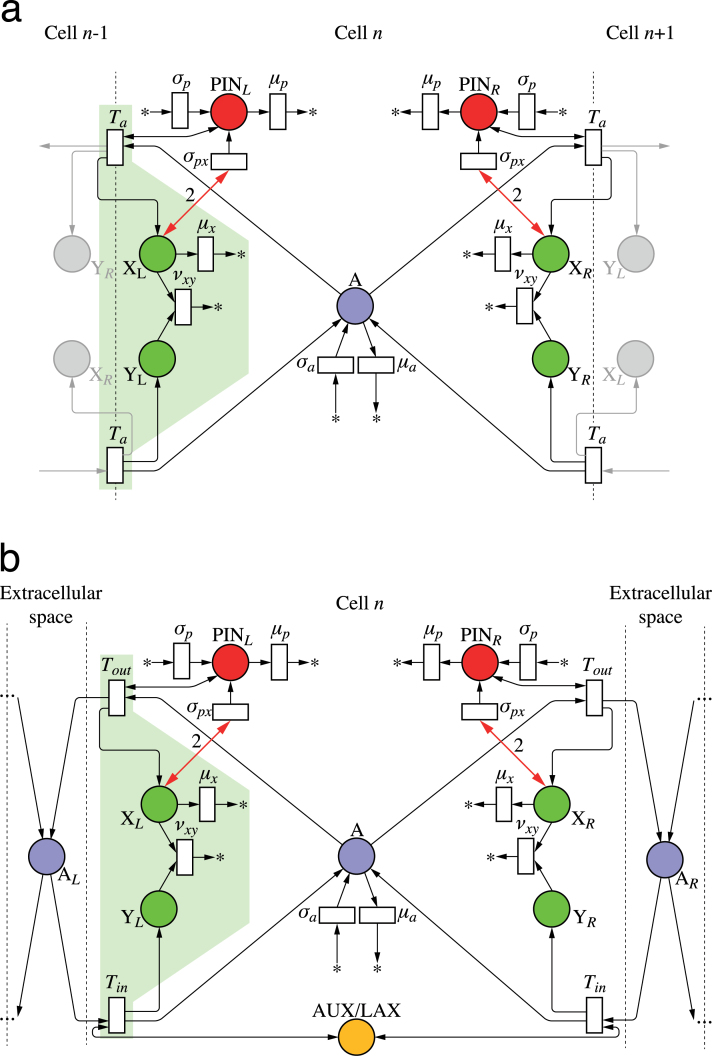
Mitchison’s cell polarization model implemented with tally molecules. Net flux through a membrane segment is measured by the circuit from Fig. 2f, highlighted for the left segment. It regulates local production of PIN by a feedback reaction (bold arrows). Extracellular space is ignored in network (a) and considered in network (b). (This figure is available in colour at *JXB* online.)

As discussed before, net fluxes can be measured by the concentration of tally molecule X in the circuit in Fig. 2f. Incorporating this circuit into a model of active auxin transport by PIN proteins yields the network depicted in Fig. 4. For clarity, this and subsequent Petri nets show only two neighbours of the cell under consideration; the extension to other numbers is straightforward. The concentration of PIN_*L*_ is the result of three reactions (analogous reactions can be written for PIN_*R*_):

$$\text{*}\overset{\sigma_{p}}{\to }{\text{PIN}}_{L}$$ (22)

$${\text{PIN}}_{L}\overset{\mu_{p}}{\to }\text{*}$$ (23)

$$2{\text{X}}_{L}\overset{\sigma_{px}}{\to }{\text{PIN}}_{L}+2{\text{X}}_{L}$$ (24)

The first two reactions represent the background production and degradation of PIN_*L*_. The third reaction represents the impact of tally molecule X_*L*_ on the production of PIN_*L*_. The concentration of PIN_*L*_ thus changes according to the equation:

$$\frac{d[PIN_{L}]}{dt}=\sigma_{px}{\left[{\text{X}}_{L}\right]}^{2}+\sigma_{p}-\mu_{p}[{\text{PIN}}_{L}]$$ (25)

By replacing [X_*L*_] with its steady-state value given by Eqn 13, and setting $\sigma_{px}=\alpha \mu_{x}^{2}$ , $\sigma_{p}=\beta$ and $\mu_{p}=\gamma$ , we obtain:

$$\frac{d[PIN_{L}]}{dt}=\{\begin{array}{ll} \alpha \phi_{L\to R}^{2}+\beta -\gamma [{\text{PIN}}_{L}] & \text{if }\phi_{L\to R}\geq 0\\ \beta -\gamma [{\text{PIN}}_{L}] & \text{if }\phi_{L\to R}<0 \end{array}$$ (26)

This equation is identical to Eqn 21 postulated by Mitchison. Note that we assumed that two X molecules are needed to add one PIN to the membrane (more precisely, we assumed that PINs are produced by a second-order reaction with respect to X, c.f. Eqn 25). If only one X molecule was used instead, flux-based PIN polarization would be a linear function of flux. Such reactions can broadly orient PIN in the direction of auxin flux, but do not lead to the formation of a canal (Stoma *et al.*, 2008; O’Connor *et al.*, 2014).

The operation of the network from Fig. 4a in a square grid of cells is shown in Supplementary Fig. S1 and Video S1 available at *JXB* online. The results are similar to those obtained by Rolland-Lagan and Prusinkiewicz (2005) using Mitchison’s model with the same parameter values. PIN concentrations in the canal are slightly higher in our simulation, because they are not clamped to a maximum value. Our results thus indicate that Mitchison’s model can potentially be implemented by a biochemically plausible network. Furthermore, the convergence of both simulations indicates that the steady-stating of tally molecule X_L_, implicitly present in Mitchison’s model, does not have a significant impact on the operation of the model.

**Fig. 5.**
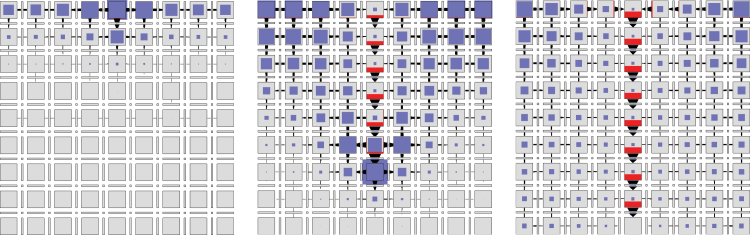
Simulation of canalization using Mitchison’s polarization model with tally molecules and extracellular space, as defined by the network in Fig. 4b. Three successive simulation stages are shown; the last stage represents the steady state. Auxin is produced by the cells in the top row. The production rate is higher in the top centre cell than in its neighbours. The cell in the centre of the bottom row is an auxin sink. (This figure is available in colour at *JXB* online.)

Both the original formulation of Mitchison’s model and its implementation by the network in Fig. 4a do not take extracellular space into account. We removed this simplifying assumption by modifying the model from Fig. 4a as shown in Fig. 4b. In the modified model, the auxin leaving a cell does not enter the neighbouring cell directly, but may accumulate and diffuse in the extracellular space. A mechanism for importing auxin into the cells is thus needed. In the net shown in Fig. 4b, this function is performed by the AUX/LAX auxin importers (Bennett *et al.*, 1996; Parry *et al.*, 2001). A tally molecule Y is assumed to be produced each time AUX/LAX imports an auxin molecule to the cell. Fig. 5 and Supplementary Video S2 (available at *JXB* online) show that the resulting canalization model works equally well in this case.

Finally, we observed that the use of the net flux is not essential to the operation of Mitchison’s model. For example, replacing the circuit for measuring net fluxes (Fig. 2f), highlighted in Fig. 4, with a circuit for measuring flux ratios (Fig. 2g) results in the network in Supplementary Fig. S2 (available at *JXB* online), which also produces a canal (Supplementary Fig. S3 and Video S3, available at *JXB* online).

#### Canalization with exo- and endocytosis

Auxin concentration in the canal emerging in Mitchison’s model is lower than in the surrounding space (Fig. 5, Supplementary Figs S1 and S3). In contrast, experimental data indicate that auxin concentration in the emerging vascular strands is relatively higher (Scarpella *et al.*, 2006). Furthermore, Mitchison’s model postulates independent production and turnover of PIN proteins in the individual sections of the membrane, whereas molecular data indicate that PINs belong to a common pool, allocated to and removed from the membranes through the dynamic processes of exo- and endocytosis (Geldner *et al.*, 2001; Vanneste and Friml, 2009). Feugier *et al.* (2005) showed that these two observations are related, and a modification of Mitchison’s model in which membranes compete for PIN allocated from a common pool produces canals with a high concentration of auxin. Similar to Mitchison, Feugier *et al.* (2005) postulated a measurement of fluxes, but did not suggest a mechanism through which it could be achieved.

**Fig. 6.**
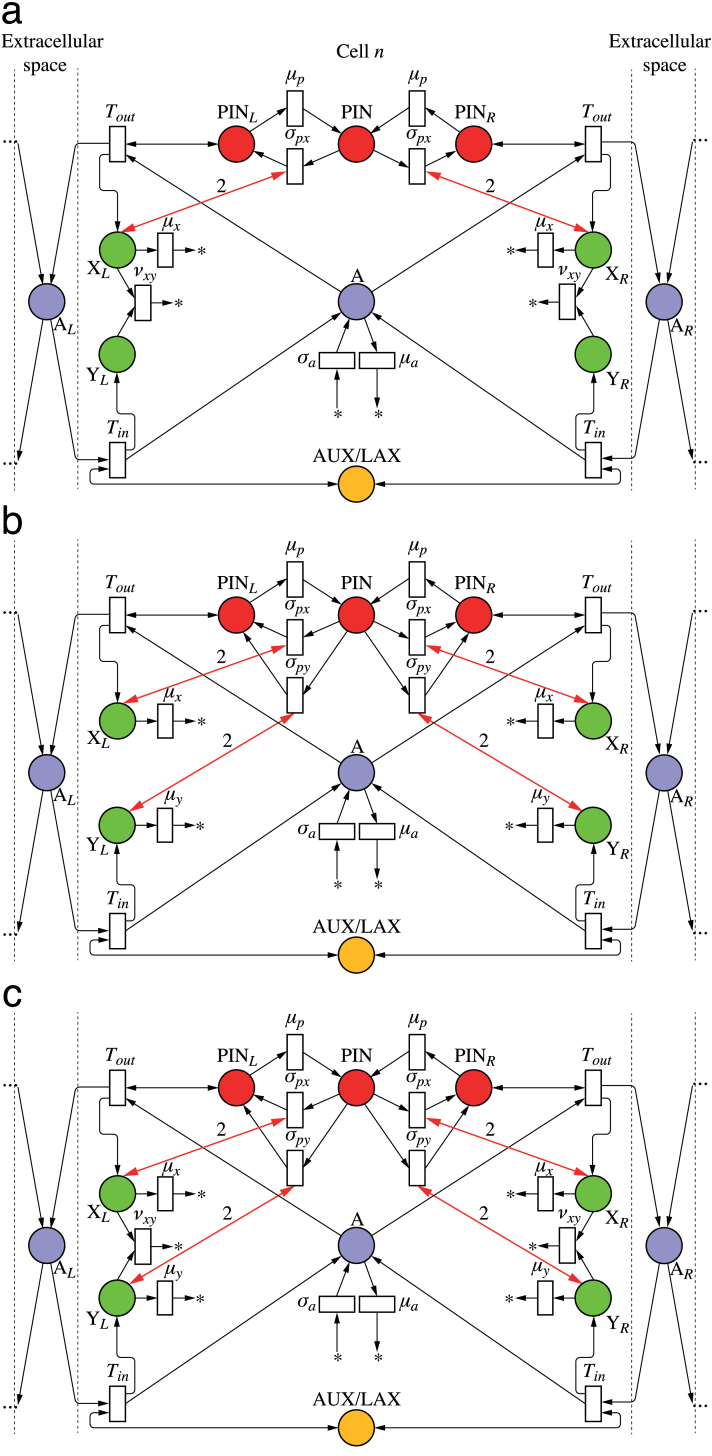
Polarization models with PIN in the membranes allocated from a pool. The networks implement canalization (a), convergence point formation (b), and dual polarization (c). (This figure is available in colour at *JXB* online.)

To show that the model of Feugier *et al.* (2005) could be implemented in a biochemically plausible manner, we modified the network of Fig. 4b by replacing the independent production and turnover of PIN protein at the membranes (Eqns 22–24) with a simulation of exo- and endocytosis (Fig. 6a; we also use this network as an example of how to convert a continuous Petri net to the equivalent set of differential equations used in simulations; see Supplementary Text S3.1, available at *JXB* online). Assuming that tally molecule X modifies the rate of exocytosis, the reactions defining the concentration of PIN allocated to the left membrane are:

$$\text{PIN}+2{\text{X}}_{L}\overset{\sigma_{px}}{\to }{\text{PIN}}_{L}+2{\text{X}}_{L}$$ (27)

$${\text{PIN}}_{L}\overset{{µ}_{p}}{\to }\text{PIN}$$ (28)

**Fig. 7.**
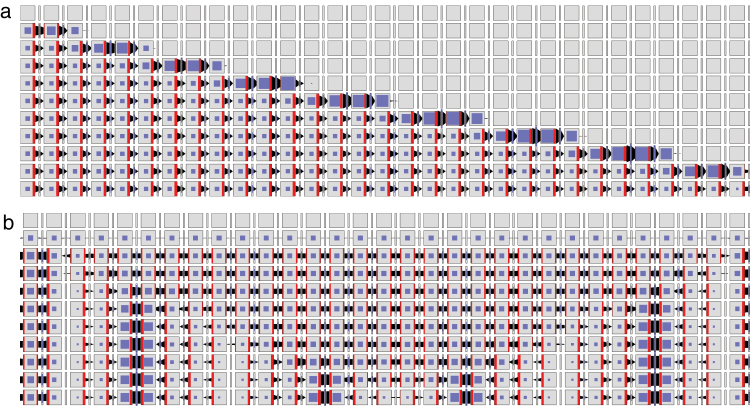
Dynamics of cell polarization in models incorporating PIN exo- and endocytosis. Consecutive rows illustrate the progress of the simulation. (a) With-the-flux polarization simulated using the network of Fig. 6a. The leftmost cell is the auxin source and the rightmost cell is the sink. (b) Convergence point formation simulated using the network of Fig. 6b. All cells produce auxin at the same rate except, to break symmetry, the leftmost cell, which produces auxin slightly faster. The leftmost and the rightmost cells are assumed to be neighbours (periodic boundary conditions). (This figure is available in colour at *JXB* online.)

In this and subsequent models, we also assume a small constitutive allocation of PIN to the membranes:

$$\text{PIN}\overset{\sigma_{p}}{\to }{\text{PIN}}_{L}$$ (29)

which, for visual clarity, is not shown explicitly in the Petri nets. The pattern of auxin and PIN distribution produced by the resulting model is shown in Supplementary Fig. S4 and Video S4, available at *JXB* online. Consistent with the results of Feugier *et al.* (2005), the emerging canal has a higher concentration of auxin than its surroundings. The dynamics of the model’s operation is further illustrated in Fig. 7a, showing the progression of cell polarization from the source to the sink over time.

#### Convergence point formation

New organs are commonly initiated at the convergence points of PIN polarization (Reinhardt *et al.*, 2003; Barbier de Reuille *et al.*, 2006). To explain the emergence of convergence points, Jönsson *et al.* (2006) and Smith *et al.* (2006) postulated models in which PIN is polarized preferentially towards neighbouring cells with the highest auxin concentration. It has been unclear, however, how cells could sense auxin concentration in their neighbours (Wabnik *et al.*, 2010; Abley *et al.*, 2013; Bennett *et al.*, 2014). Sahlin *et al.* (2009) considered a modification in which auxin in the extracellular space, rather than in the neighbouring cells, is the source of feedback. They showed that such a model creates a sequence of cells with alternating polarities rather than a pattern of spatially separated convergence points. Pursuing a different approach, Stoma *et al.* (2008) proposed a flux-based model of convergence point formation that exploited the continuity of auxin flow towards the convergence point (in the epidermis) and away from it (in the subepidermal tissues). This model produces patterns of PIN polarization consistent with experimental data, but the initial auxin concentration at the convergence points is lower than in the surrounding tissue in an apparent disagreement with the data.

The shortcomings of previous models are avoided if PIN is allocated to the membrane segments in response to a weighted sum of auxin influx (which can be viewed as a measure of auxin concentration in the extracellular space, Eqn 4) and efflux. The proposed network is shown in Fig. 6b. It modifies the network from Fig. 6a in two points. First, the tally molecules X and Y measure the efflux and influx of auxin through a membrane independently, as in the circuit of Fig. 2c. Secondly, both molecules promote exocytosis:

$$\text{PIN}+2{\text{X}}_{L}\overset{\sigma_{px}}{\to }{\text{PIN}}_{L}+2{\text{X}}_{L}$$ (30)

$$\text{PIN}+2{\text{Y}}_{L}\overset{\sigma_{py}}{\to }{\text{PIN}}_{L}+2{\text{Y}}_{L}$$ (31)

**Fig. 8.**
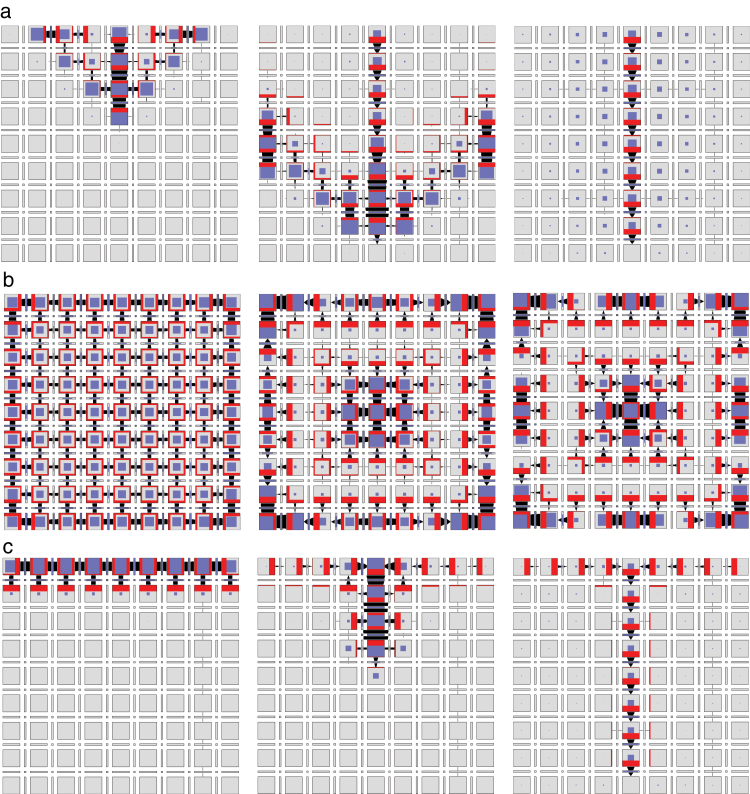
Patterns generated using the network from Fig. 6c. Three successive simulation stages are shown. (a) Canalization. The cell in the centre of the top row of cells is an auxin source. The cell in the centre of the bottom row is an auxin sink. (b) Convergence point formation. All cells have the same parameters. (c) Dual polarization. The grid can be interpreted as a longitudinal section of a portion of a shoot apical meristem near an emerging primordium. The cells in the top row represent the epidermis and produce auxin at a higher rate than the remaining cells, which represent inner tissues. The cell in the centre of the bottom row is an auxin sink. The simulation begins with the auxin concentration in all cells set to zero. A convergence point emerges in the centre cell of the top row and then propagates into the inner tissue creating a canal of high auxin concentration. The transition from up-the-gradient to with-the-flux mode is controlled by auxin concentration in the cell, which affects the annihilation rate *v*_*xy*_ of the tally molecules. (This figure is available in colour at *JXB* online.)

The operation of this model in a two-dimensional grid of cells is shown in Fig. 8b and Supplementary Video S6, available at *JXB* online. The dynamics of pattern formation is further illustrated using a one-dimensional file of cells in Fig. 7b. In both cases, the model produces a regular pattern of approximately equidistant convergence points. Their distance is easily controlled by the rate of auxin transport. Incidentally, this distance is positively correlated with the rate of auxin transport (Supplementary Fig. S5, available at *JXB* online), in contrast to the original models of up-the-gradient polarization, where it decreases as the rate of transport increases (see Fig. 6c in Runions *et al.*, 2014).

#### Dual polarization

At convergence points, the processes of convergence point formation and canalization co-exist. To explain this phenomenon, Bayer *et al.* (2009) proposed the dual polarization model. According to this model, an up-the-gradient polarization mechanism underlying convergence point formation and a with-the-(net)-flux mechanism underlying canalization act simultaneously within a cell. Bayer *et al.* (2009) also suggested that the proportions in which these mechanisms are combined is regulated by auxin concentration within each cell. The dynamic patterns of PIN polarization and auxin distribution produced by the dual polarization model are consistent with experimental data, but the assumed co-existence of two different polarization mechanisms has been questioned (Merks *et al.*, 2007; Stoma *et al.*, 2008).

To examine whether a single network can act in both polarization modes, we integrated the Petri nets in Fig. 6a and b into a single net in Fig. 6c. The turnover of the tally molecules X and Y is thus defined by the union of reactions shown in Fig. 6a and b:

$$X_{L}\overset{\mu_{x}}{\to }*$$ (32)

$$Y_{L}\overset{\mu_{y}}{\to }*$$ (33)

$$X_{L}+Y_{L}\overset{\nu_{xy}}{\to }*$$ (34)

and both tally molecules modify the rate of PIN exocytosis as in Fig. 6b (Eqns 30 and 31). The steady-state concentration of molecules X and Y in this network is described by a quadratic equation (Eqn S12 in Supplementary Text S3.2), which does not have as straightforward an interpretation as the steady-state solutions of models discussed so far (see Eqns S13 and S15 in Supplementary Text S3.2). It is evident, however, that if *v*_*xy*_=0 (i.e. when X and Y cannot annihilate each other), the network of Fig. 6c is identical to the network of Fig. 6b, and thus capable of the formation of convergence points (Fig. 8b). Moreover, simulations show that increased annihilation rates *v*_*xy*_ switch the behaviour of the network in Fig. 6c towards that of the network in Fig. 6a, producing canals (Fig. 8a and Supplementary Video S5, available at *JXB* online). The network of Fig. 6c can thus transition between with-the-flux and up-the-gradient modes with the change of a single reaction rate.

To examine whether this network can also simulate the integrated emergence of convergence points and canals postulated by the dual polarization model, we assumed that the coefficient *v*_*xy*_ of tally molecule annihilation switches from low to high when auxin concentration increases above a threshold (*a*_*th*_ in Supplementary Table S1). The resulting simulation is illustrated in Fig. 8c and Supplementary Video S7, available at *JXB* online. When auxin is present at low to medium levels, a convergence point emerges in the epidermis. An increasing auxin concentration in the cell at the convergence point eventually switches it to the canalization mode. The auxin maximum then propagates into the inner tissue, leaving behind a canal that eventually reaches the sink in the middle of the bottom row (an additional sink-finding mechanism would be needed to reach an arbitrarily positioned sink; Bayer *et al.*, 2009; O’Connor *et al.*, 2014). In the early stages of the simulation, the subepidermal cells are polarized towards the epidermis, and at later stages, cells on both sides of the canal are polarized towards it. The emerging pattern is thus consistent with the experimental evidence that led to the formulation of the dual polarization model (Bayer *et al.*, 2009).

### Diverse modes of flux measurement make room for a wide range of models

In the networks considered so far, we assumed that tally molecules X and Y measure unidirectional auxin fluxes and combine the measurement results via simple circuits. However, unidirectional fluxes can also be measured through the concentration of auxin transporters (see Section ‘Concentration of transporters’) or auxin itself (see Section ‘Concentration of transported substance’). We show that polarization models in which these concentrations feed back on exocytosis and/or endocytosis can produce convergence points and canals of auxin transport as well.

#### Transport may be regulated by auxin-bound transporters

**Fig. 9.**
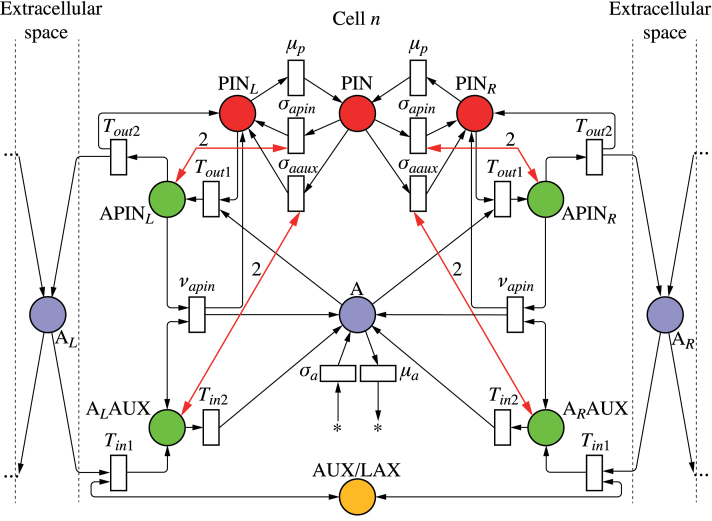
Polarization model in which auxin fluxes are measured by the concentrations of effux and influx carriers binding auxin in the membrane (APIN and AAUX). (This figure is available in colour at *JXB* online.)

**Fig. 10.**
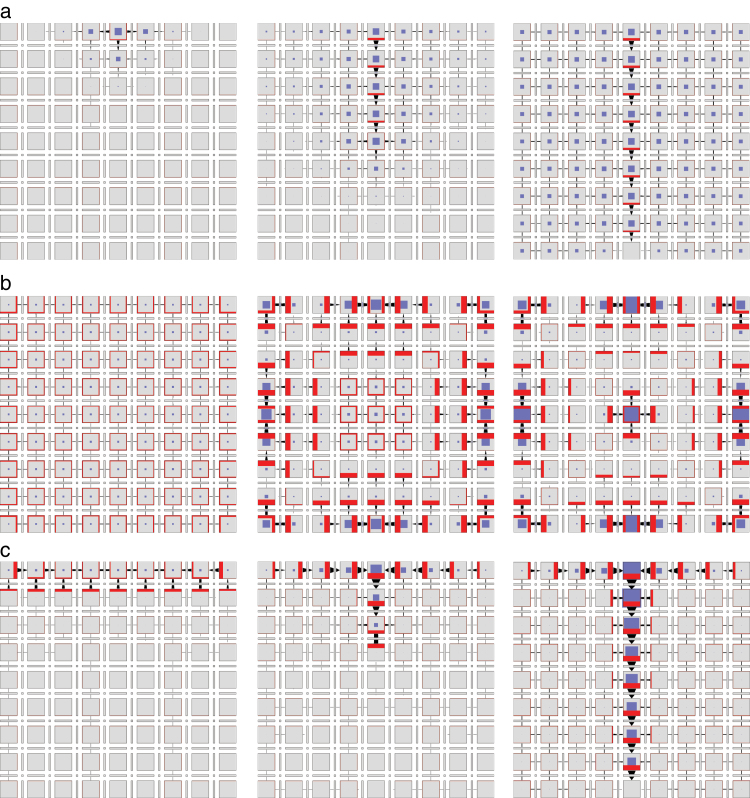
Dynamics of cell polarization by the network in Fig. 9. Depending on the value of reaction rate constant *v*_*apin*_, the network simulates canalization (a, *v*_*apin*_>0), convergence point formation (b, *v*_*apin*_≈0), or convergence point formation combined with canalization according to the dual polarization model (c, *v*_*apin*_ is controlled by the concentration of auxin in each cell). The initial conditions in each simulation are qualitatively the same as in Fig. 8. (This figure is available in colour at *JXB* online.)

To explore the patterning potential of models in which fluxes are measured by concentrations of auxin-bound carriers, we first investigated a simple network in which these concentrations affect directly the rate of PIN exocytosis (Fig. 9). In essence, the intermediate molecules APIN_*L*_ and A_*L*_AUX then play the role of tally molecules X and Y from Fig. 6. With *v*_*apin*_=0, the network in Fig. 9 has a structure analogous to the network in Fig. 6b, and both networks produce convergence points in a similar manner (compare Figs 10b and 8b, as well as Supplementary Videos S9 and S6, available at *JXB* online). If *v*_*apin*_ > 0, the complex A_*L*_AUX breaks down the complex APIN_*L*_ according to the reaction:

$${\text{A}}_{L}\text{AUX}+{\text{APIN}}_{L}\overset{\nu_{apin}}{\to }{\text{A}}_{L}\text{AUX}+\text{A}+{\text{PIN}}_{L}$$ (35)

and the interaction between A_*L*_AUX and APIN_*L*_ has the structure shown in Fig. 2g. The concentration of APIN_*L*_ thus measures the ratio of auxin outflux to influx, and its feedback on exocytosis leads to canalization of auxin fluxes (Fig. 10a and Supplementary Video S8, available at *JXB* online). This result is consistent with the modification of Mitchison’s model, in which concentration of auxin export carriers depends on the flux ratio rather than the net flux (see Section ‘Mitchison’s canalization model’). Finally, similar to the two-tally-molecule network of Fig. 6c, the network of Fig. 9 can simulate the emergence of a convergence point and the formation of a midvein consistent with the dual polarization model (Fig. 10c and Supplementary Video S10, available at *JXB* online). As in the simulation using tally molecules (Fig. 8c), we assumed that the reaction rate constant *v*_*apin*_ was a step function of auxin concentration in the cell.

#### Bound transporters may feed back on PIN localization via an intermediate molecule

Fig. 9 implies that auxin efflux and influx carriers bound to auxin (APIN and AAUX) directly influence the rate of exocytosis, and directly interact with each other. At present, these assumptions have no experimental support. Consequently, we also constructed hypothetical networks in which the interactions between APIN and AAUX, and their feedback on the allocation of PIN to the membrane, are mediated by an additional molecule.

We based these networks on the circuit in Fig. 2i, which measures the combined ratio of the sum of unidirectional fluxes (see Section ‘Ratio of weighted sums of fluxes’). The production and annihilation of the mediating molecule Z_*L*_ is catalysed by APIN_*L*_ and A_*L*_AUX according to the reactions:

$${\text{APIN}}_{L}\overset{k_{1}}{\underset{k_{2}}{↔}}{\text{APIN}}_{L}+{\text{Z}}_{L}$$ (36)

$${\text{A}}_{L}\text{AUX}\overset{k_{3}}{\underset{k_{4}}{↔}}\;{\text{A}}_{L}\text{AUX}+{\text{Z}}_{L}$$ (37)

In addition, $Z_{L}$ is constitutively produced and turned over:

$$\text{*}\overset{\sigma_{z}}{\to }{\text{Z}}_{L}$$ (38)

$$Z_{L}\overset{\mu_{z}}{\to }*$$ (39)

By substituting APIN_*L*_ for A_*LR*_ and A_*L*_AUX for A_*RL*_ in Eqn 18, we obtain:

$$[{\text{Z}}_{L}]=\frac{k_{1}[APIN_{L}]+k_{3}[A_{L}AUX]+\sigma_{z}}{k_{2}[APIN_{L}]+k_{4}[A_{L}AUX]+\mu_{z}}$$ (40)

or, in terms of fluxes:

$$[Z_{L}]=\frac{\kappa_{1}J_{A\to A_{L}}+\kappa_{3}J_{A_{L}\to A}+\sigma_{z}}{\kappa_{2}J_{A\to A_{L}}+\kappa_{4}J_{A_{L}\to A}+\mu_{z}}$$ (41)

where $\kappa_{1}=k_{1}/T_{out2}$ , $\kappa_{2}=k_{2}/T_{out2}$ , $\kappa_{3}=k_{3}/T_{in2}$ , $\kappa_{4}=k_{4}/T_{in2}$ (c.f. Section ‘Ratio of weighted sums of fluxes’).

**Fig. 11.**
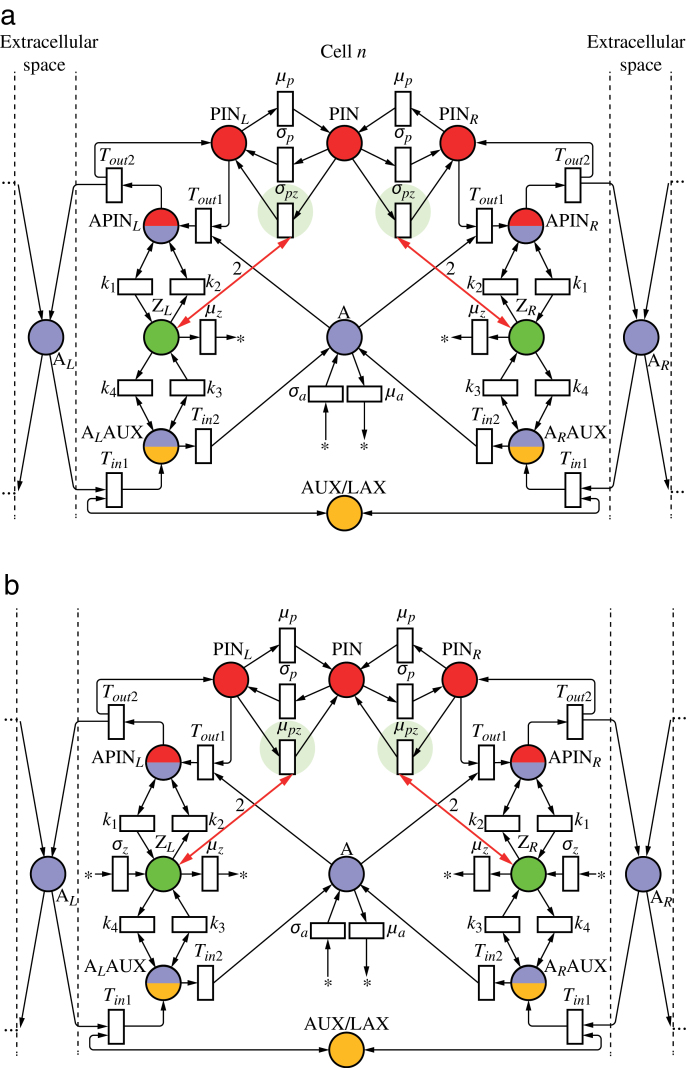
Two polarization networks in which auxin fluxes are measured by auxin transporters and feed back on PIN localization via an intermediate molecule. The molecule controls exocytosis (a) or endocytosis (b) of PIN proteins. The small difference between both networks is highlighted by pale shaded circles. (This figure is available in colour at *JXB* online.)

We considered two models in which PIN allocation to a membrane is controlled by the mediating molecule Z. In the first model, shown in Fig. 11a, Z promotes PIN exocytosis:

$$2{\text{Z}}_{L}+\text{PIN}\overset{\sigma_{pz}}{\to }2{\text{Z}}_{L}+{\text{PIN}}_{L}$$ (42)

**Fig. 12.**
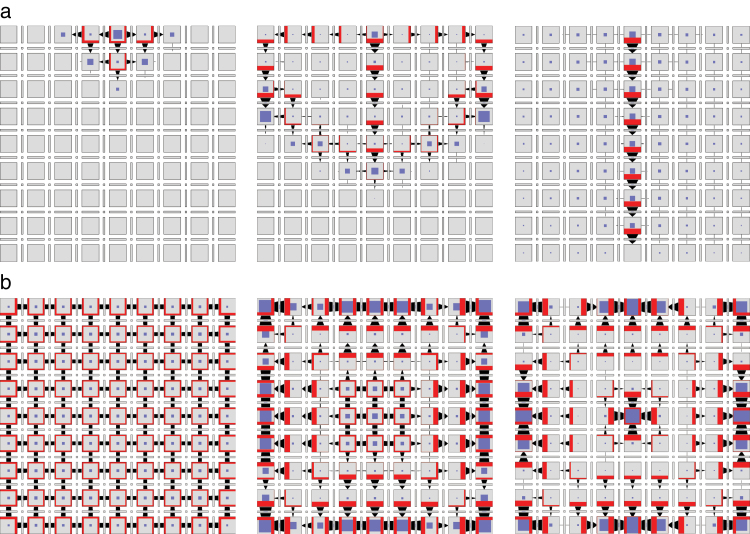
Dynamics of cell polarization in the network of Fig. 11a. Depending on the value of reaction constant *k*_4_, the network simulates canalization (a, *k*_4_=0.5) or convergence point formation (b, *k*_4_=0.01). The initial conditions in each simulation are qualitatively the same as in Fig. 8. (This figure is available in colour at *JXB* online.)

Our previous models indicated that the control of exocytosis by the ratio of influx to efflux leads to canalization (see Section ‘Mitchison’s canalization model’), and the control by a weighted sum of fluxes leads to the generation of convergence points (see Section ‘Convergence point formation’). Both flux measures can easily be approximated by setting $k_{2}=\sigma_{z}=0$ and $k_{1}>k_{3}$ in Eqn 41. Simulations show that the model switches from generating a canal (Fig. 12a and Supplementary Video S11, available at *JXB* online), to generating convergence points (Fig. 12b and Supplementary Video S12, available at *JXB* online) with the change of a single reaction constant (*k*_4_).

In the alternative model, shown in Fig. 11b, Z promotes PIN endocytosis:

$$2{\text{Z}}_{L}+{\text{PIN}}_{L}\overset{\mu_{pz}}{\to }2{\text{Z}}_{L}+\text{PIN}$$ (43)

**Fig. 13.**
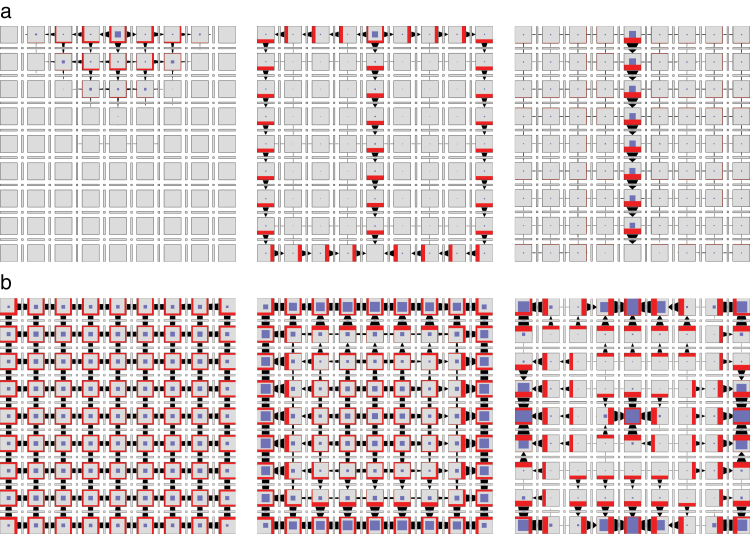
Dynamics of cell polarization for the networks in Figs. 11b and 14. Depending on the value of reaction constant *k*_3_, these networks simulate canalization (a, *k*_3_=0.5) or convergence point formation (b, *k*_3_= 0.01). The initial conditions in each simulation are qualitatively the same as in Fig. 8. Symmetry breaking in case (b) is due to limited precision of floating-point arithmetic. (This figure is available in colour at *JXB* online.)

In this case, we set $k_{1}=0$ and $\sigma_{z}=1$ , so that the auxin outflux promotes accumulation of PIN in the membrane by inhibiting endocytosis. Simulations show that, with $k_{2}>k_{4}$ , the model can again switch between generating a canal (Fig. 13a and Supplementary Video S13, available at *JXB* online) and convergence points (Fig. 13b and Supplementary Video S14, available at *JXB* online) with the change of a single reaction rate (*k*_3_). Thus, the regulation of auxin abundance in the membrane through endocytosis produces similar results to the regulation through exocytosis.

#### Extracellular auxin concentration can be sensed in lieu of auxin influx

**Fig. 14.**
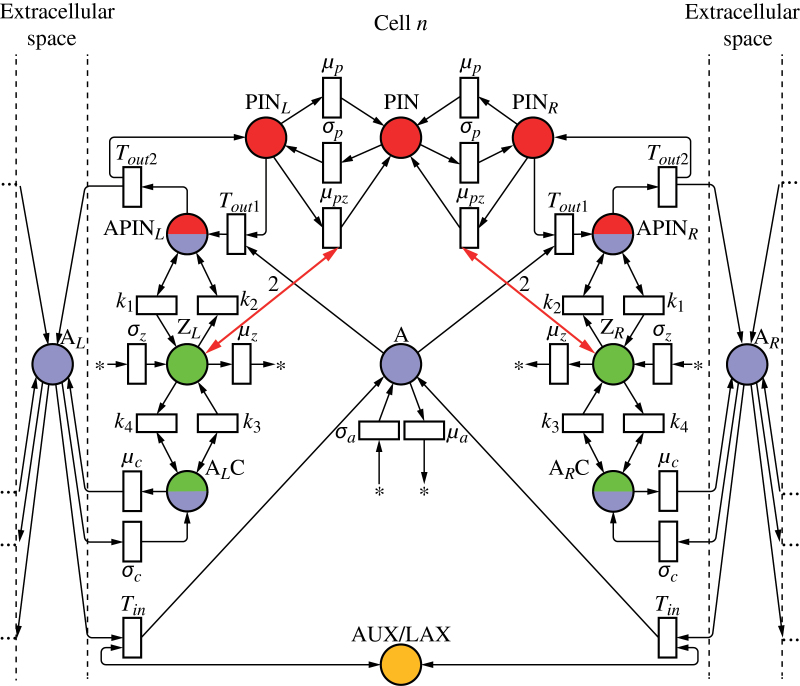
Variant of the model from Fig. 11b, in which sensing of extracellular auxin is decoupled from the measuring of auxin influx. (This figure is available in colour at *JXB* online.)

In the models of Figs 9 and 11, the concentration of auxin influx carriers bound to auxin (A_*L*_AUX) provides a measure of both the auxin influx and its concentration in the extracellular space. A variant of the model from Fig. 11b, in which the concentration of extracellular auxin is measured independently from the auxin influx, is shown in Fig. 14. The role of complex A_*L*_AUX in sensing extracellular auxin is now assumed by a hypothetical membrane-localized complex A_*L*_C, which has the steady-state concentration proportional to the extracellular auxin concentration:

$$\left[A_{L}C]=\frac{\sigma_{c}}{\mu_{c}}[A_{L}\right]$$ (44)

The essential elements of the network in Fig. 14 are thus: a positive feedback of auxin efflux on the membrane allocation of PIN (reaction with constant *k*_2_), and a further control of this allocation by auxin concentration in the extracellular space (reactions with constants *k*_3_ and *k*_4_). Depending on whether increased auxin concentration in the extracellular space decreases or increases PIN allocation to the corresponding portion of the cell membrane, the network generates canals or convergence points. Simulation results are identical to those obtained for the network in Fig. 11b, and shown in Fig. 13 and Supplementary Videos S13 and S14.

## Discussion

Auxin fulfils an essential patterning role in plant development, but many aspects of its action have remained unclear. Fundamental questions concern both molecular-level mechanisms leading to the polarization of individual cells and their integration into patterning processes taking place at the level of entire tissues. As a step towards addressing these questions, we proposed that a cell measures unidirectional auxin fluxes that enter and leave it through different segments of the membrane. We have shown that such fluxes and their combinations can be monitored by simple biochemical circuits, and we employed this observation to propose plausible implementations of selected polarization models, both described in the literature and new. To describe these models, we employed the formalism of Petri nets. Petri nets provide an intuitive yet precise graphical method for specifying reaction and transport networks, which facilitates the definition and comparisons of different networks that may be involved in auxin-driven patterning. These networks are inherently consistent with the law of mass action and thus meet the fundamental requirement of biochemical plausibility.

In principle, transport-driven patterning may occur by auxin controlling the effectiveness of efflux or influx of auxin transport (Runions *et al.*, 2014). Biological data indicate that auxin efflux carriers, in particular PIN allocated to the cell membrane, are the primary target of dynamic transport control (Paciorek *et al.*, 2005; Wiśniewska *et al.*, 2006). Consistent with the canalization theory (Sachs, 1969, 1991), our models require a positive feedback of auxin efflux on the abundance or effectiveness of auxin efflux carriers. However, modulation of this feedback by auxin influx (which may be measured in different ways, including the concentration of auxin in the extracellular space) is also necessary, as it is the influx that provides the cell with the information about its surroundings.

**Fig. 15.**
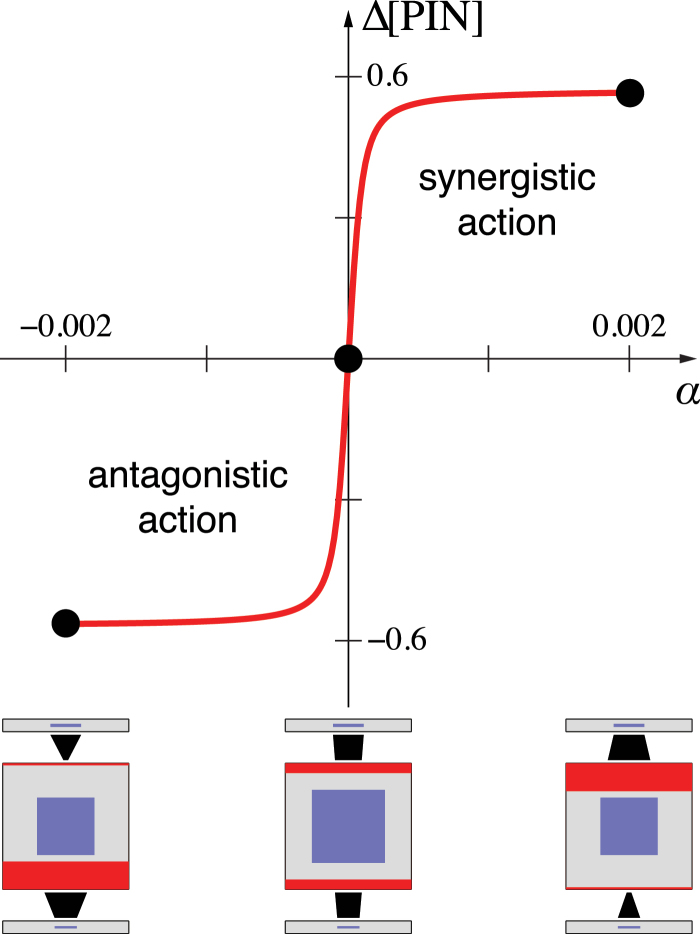
Contrasting roles of auxin influx in cell polarization. The cell is placed between two extracellular compartments with predefined auxin concentrations. The concentration in the top compartment is slightly higher than in the bottom compartment. PIN exocytosis is controlled at each membrane by a linear combination of outflux and influx, *J*_out_+*αJ*_in_ (see Supplementary Text S3.3 for model details). Cell polarization is measured as the difference Δ[PIN] in PIN concentration at the top and the bottom membrane. Steady-state cell polarization for three data points is shown below the graph. The cell polarizes towards the extracellular compartment with lower auxin concentration if the outflux and influx act antagonistically (*α*<0), remains unpolarized if *α*=0, and polarizes towards the compartment with a higher auxin concentration if the outflux and influx act synergistically (*α*>0). The switch between both polarization types occurs within a small range of parameter values (*α*≈0). (This figure is available in colour at *JXB* online.)

Analysis of the presented models indicates that the influx affects PIN polarization in a predictable manner, with two basic behaviours. If the influx has an opposite effect to efflux, antagonistically reducing the concentration of auxin efflux carriers in the membrane, PIN in the cell is preferentially allocated to the membrane adjacent to the extracellular compartment with lower auxin concentration. If, in contrast, influx acts in the same manner as the efflux, synergistically increasing the concentration of auxin efflux carriers, PIN becomes polarized towards the compartment with higher auxin concentration. We confirmed these contrasting behaviours with a simple model of an isolated cell, in which PIN polarization is controlled by a linear combination of outflux and influx (Fig. 15; see Supplementary Text S3.3 for model details).

Extensions of the above model to a file of cells (Supplementary Text S3.4) provide additional insights into the dynamics of pattern formation in the antagonistic versus synergistic polarization regimes. Antagonistic action of efflux and influx leads to a wave of cell polarization that propagates away from the cell with higher initial auxin concentration. This may lead to the formation of a PIN convergence point and auxin maximum at the location most distal to the initial cell (Supplementary Fig. S7 and Video S15, available at *JXB* online). Additional convergence points and auxin maxima may arise in the presence of noise (results not shown). In contrast, synergistic polarization leads to the formation of a PIN convergence point in the cell that initially had higher auxin concentration than its neighbours (Supplementary Fig. S9 and Video S16, available at *JXB* online). Depending on the parameters of the system, additional maxima may subsequently emerge (Supplementary Fig. S11, available at *JXB* online). The phenomena commonly referred to as with-the-flux and up-the-gradient polarization may thus be explained by a common mechanism, in which the initial polarization is determined by the influence of auxin influx, and the steady state is maintained by a positive feedback of auxin efflux on PIN polarization. The distinction between both polarization modes is thus reduced to the sign of the initial influence of the influx. The steady state is always with the flux.

The hypothesis of polarization by unidirectional fluxes sheds light on the experimental results that so far did not find a fully satisfactory explanation. Of central morphogenetic importance are patterns of PIN polarization and auxin flow that lead to the formation of a convergence point and a canal of auxin transport originating at this point (Reinhardt *et al.*, 2003; Scarpella *et al.*, 2006; Hay *et al.*, 2006). Experimental results and observations are consistent with the dual polarization model (Bayer *et al.*, 2009), which postulated coexistence and coordinate action of up-the-gradient and with-the-flux polarization models. This explanation did not appear to be parsimonious, however, and alternative models have been sought (Merks *et al.*, 2007; Stoma *et al.*, 2008). We have shown that the switch from synergistic to antagonistic polarization regime suffices to coordinate the formation of a convergence point and canal, and such a switch my result from the change in a single reaction rate. The logic of dual polarization is thus plausible.

A similar switch may explain the reversal of PIN polarization accompanying mutation or overexpression of the *PINOID* gene (Friml *et al.*, 2004). This reversal is a non-intuitive phenomenon, as it is easier to conceptualize changes in the degree of polarization (Mattsson *et al.*, 1999; Rolland-Lagan and Prusinkiewicz, 2005), or even a complete destruction of cell polarity in a mutant, than a coordinated change of the polarity direction due to a non-directional signal (the expression of *PINOID*). A mechanism in which PINOID—acting, possibly, in conjunction with the MAB4 proteins (Furutani *et al.*, 2014) or calcium ions (Zhang *et al.*, 2011)—would affect PIN polarization by controlling the deployment of antagonistic versus synergistic PIN allocation regimes offers a plausible explanation of the experimental results.

A characterization of the morphospace of generated patterns—extending the mathematical analyses pioneered by van Berkel *et al.* (2013), Draelants *et al.* (2013), Farcot and Yuan (2013), and Feller *et al.* (2014)—would be useful in assessing whether models postulating the measurement of unidirectional fluxes of auxin can explain the full range of auxin-driven patterns observed in nature. For example, Hertel and Flory (1968) observed oscillations in auxin transport rates following the application of exogenous auxin to an auxin-depleted tissue, and it would be informative to verify whether they can be reproduced by the models.

The models presented in this paper separate three aspects of auxin-dependent patterning: polar auxin transport, the measurement of auxin fluxes or concentrations, and the impact of these measurements on the allocation of PIN to the membrane. It is an open question whether changes in the parameter values affecting individual aspects of these models may lead to distinct behaviours that could be correlated with experimental data. For example, would impediment of auxin sensing, PIN allocation, or auxin transport capabilities lead to different effects in the group of phenomena broadly characterized as inhibition of polar transport? What is the spectrum of parameter changes that could capture the differences between weak and strong polarization models postulated by Stoma *et al.* (2008), and the different polarization properties of the *Brachypodium* proteins SoPIN1, PIN1a, and PIN1b postulated by O’Connor *et al.* (2014)? Can the experimental results pertinent to the mutation or overexpression of the D6 PROTEIN KINASE (D6PK) (Barbosa and Schwechheimer, 2014), which affects auxin transport by PIN without changing its polarity, be explained in terms of the modulation of auxin transport efficiency (parameter *T*_out_) in our models? Answers to these questions could contribute to the validation of the models and the focus of future experiments.

A further question is whether the measurement of fluxes, if indeed present, is accomplished by tally molecules distinct from the transporters (see Sections ‘Canalization with exo- and endocytosis’, ‘Convergence point formation’, and ‘Dual polarization’), or whether PIN and/or AUX/LAX proteins are ‘transceptors’ (Kriel *et al.*, 2011), and both transport and sense auxin (see Sections ‘Transport may be regulated by auxin-bound transporters’ and ‘Bound transporters may feed back on PIN localization via an intermediate molecule’). The latter possibility was suggested by Hertel (1995) (see also Hertel and Flory, 1968), who proposed that ‘the IAA efflux carrier—or a close homologue—is the functional receptor, directly involved in auxin action.’ In this context, it may be useful to re-examine existing experimental results involving different PIN or AUX/LAX proteins, and find out whether their individual properties may be attributed to, and explained in terms of, different auxin sensing, rather than transporting, capabilities.

In the domain of influx carriers, Stieger *et al.* (2002) reported that plants grown under the influence of naphthoxyacetic acid (NOA) have a similar phenotype (defective patterning of organ primordia in the shoot apical meristem) to plants grown with naphthaleneacetic acid (NAA). NOA reportedly acts as an inhibitor of auxin influx, whereas NAA can enter a cell without the assistance of influx carriers (Delbarre *et al.*, 1996; Stieger *et al.*, 2002). The similarity of responses points to a possible role of AUX/LAX in the measurement of auxin influx: although NAA can enter cells, bypassing the AUX/LAX path may imply that the influx of NAA is not measured, producing an effect comparable to a decrease or absence of influx. It would be informative to repeat these experiments using 2-NOA and 3-chloro-4-hydroxyphenylacetic acid (CHPAA), which according to Laňková *et al.* (2010) appear to act more exclusively on the active influx than 1-NOA used by Steiger *et al.* (2002).

Alternatively, it is possible that auxin fluxes are measured by tally molecules separate from the auxin carriers, or that auxin concentration in the extracellular space is measured in lieu of auxin influx (see Section ‘Extracellular auxin concentration can be sensed in lieu of auxin influx’). The ABP1 protein was considered a likely candidate for such a concentration sensor (reviewed by Grones and Friml, 2015, for example), but Gao *et al.* (2015) recently presented arguments against this role for ABP1 by showing that *abp1* mutants do not have clear phenotypes. Another group of potential auxin sensors are ROP proteins (Xu *et al.*, 2010; Abley *et al.*, 2013). As in the case of the transceptor hypothesis, a re-examination of the existing experimental results may shed light on whether ROPs would better fit the role of flux or concentration sensors. Protons are also a potential candidate for a tally molecule, as the protonated auxin from the extracellular space disassociates into auxin ions (IAA^–^) and protons (H^+^) upon entering a cell, whereas IAA^–^ exported from the cell associates with a proton. Consequently, the concentration of protons (and thus pH) near the membrane may provide an indication of the net flux (Steinacher *et al.*, 2012). A feedback of pH on auxin import by AUX1 has been reported by Cho *et al.* (2012), and it would be informative to consider whether pH may feed back on PIN allocation as well. Even if such feedback is present, however, our results would suggest that an additional input is needed, as the measurement of net fluxes does not suffice to implement the synergistic polarization mode.

In addition to the pivotal role played by the relatively well-characterized auxin carriers of the PIN and AUX/LAX families, recent experimental work has highlighted a number of additional processes that play a role in auxin-based patterning. These include: (i) the regulation of auxin availability, via synthesis, turnover, and conversion to and from conjugated forms (Korasick *et al.*, 2013); (ii) ATP-driven auxin transport via ABCB proteins (Zažímalová *et al.*, 2010); (iii) passive auxin fluxes, possibly through the plasmodesmata (Mitchison, 1980), which could play an essential role in PIN-independent auxin-based patterning (Guenot *et al.*, 2012; Kierzkowski *et al.*, 2013); (iv) complex interactions between auxin and internal cell components (Luschnig and Vert, 2014); and (v) biomechanical factors involved in auxin patterning (Hamant *et al.*, 2008). At present, an understanding of the biological importance of these processes, compared with the hypothesized feedbacks between auxin and PIN polarity considered in the current study, is only beginning to emerge. Consequently, an intriguing direction for future work is the use of the models proposed here as components of more comprehensive models, in which nuanced feedbacks between diverse auxin-related polarization and patterning mechanisms could be interrogated computationally.

In conclusion, the models presented in this paper demonstrate that: (i) diverse polarization models can potentially be implemented by molecularly plausible mechanisms; (ii) the polarization modes so far attributed to separate up-the-gradient and with-the-flux mechanisms can be implemented by a common mechanism; (iii) the deployment of either polarization mode may depend on as little as a single reaction rate; (iv) both polarization modes can be understood in terms of antagonistic versus synergistic action of unidirectional auxin fluxes; and (v) the switch between antagonistic and synergistic polarization regimes provides a parsimonious explanation for experimental data including the formation of convergence points and veins, and polarization reversal. Polarization by unidirectional fluxes thus offers a conceptual framework, in which re-examination of existing experimental results, and new experimental results guided by the models, may lead to a better understanding of auxin-driven patterning in nature.

## Supplementary data

Supplementary data are available at *JXB* online. Simulation parameters are provided in Tables S1 and S2. Simulation results not included in the main text are presented in Figs S1–S5. Almost all simulations are illustrated by Videos S1–S14. The supplementary data also includes Supplementary Text S3 complemented by Table S3, Figs S6–S11, and Videos S15 and S16, which provide additional analysis and explanation of the results in the main text.

Table S1. Parameter values used in simulations illustrated in the main text.

Table S2. Parameter values used in supplementary simulations.

Table S3. Parameter values, initial conditions and boundary conditions used in the simplified model of cell polarization shown in Fig. 15.

Fig. S1. Simulation of canalization using Mitchison’s model implemented with tally molecules.

Fig. S2. A variant of Mitchison’s model with tally molecules and extracellular space, in which PIN allocation is controlled by the ratio of unidirectional fluxes.

Fig. S3. Simulation of canalization using the variant of Mitchison’s model defined by the Petri net in Fig. S2.

Fig. S4. Simulation of canalization with exocytosis controlled by tally molecules.

Fig. S5. Simulations of convergence point formation with exocytosis controlled by tally molecules.

Fig. S6. Extended visualization of a cell.

Fig. S7. Dynamics of pattern formation in a file of cells with PIN polarized by auxin efflux and influx acting antagonistically.

Fig. S8. Pattern formation in a file of cells with PIN polarized by auxin efflux and influx acting antagonistically.

Fig. S9. Dynamics of pattern formation in a file of cells with PIN polarized by auxin efflux and influx acting synergistically.

Fig. S10. Pattern formation in a file of cells with PIN polarized by auxin efflux and influx acting synergistically.

Fig. S11. Convergence points produced in cell files of different length.

Video S1. Canalization using Mitchison’s model implemented with tally molecules.

Video S2. Canalization using Mitchison’s model with tally molecules and extracellular space.

Video S3. Canalization using a variant of Mitchison’s model with tally molecules and extracellular space, in which PIN allocation is controlled by the ratio of unidirectional fluxes instead of the net flux.

Video S4. Canalization with exocytosis controlled by tally molecules.

Video S5. Canalization with exocytosis controlled by tally molecules.

Video S6. Convergence point formation with exocytosis controlled by tally molecules.

Video S7. Dual polarization with exocytosis controlled by tally molecules.

Video S8. Canalization with exocytosis controlled by influx and efflux carriers bound to auxin in the membrane.

Video S9. Convergence point formation with exocytosis controlled by influx and efflux carriers bound to auxin in the membrane.

Video S10. Dual polarization with exocytosis controlled by influx and efflux carriers bound to auxin in the membrane.

Video S11. Canalization with exocytosis controlled by influx and efflux carriers that act via a mediating molecule.

Video S12. Convergence point formation with exocytosis controlled by influx and efflux carriers that act via a mediating molecule.

Video S13. Canalization with endocytosis controlled by influx and efflux carriers that act via a mediating molecule.

Video S14. Convergence point formation with endocytosis controlled by influx and efflux carriers that act via a mediating molecule.

Video S15. Dynamics of pattern formation in a file of cells, with PIN polarized by auxin efflux and influx acting antagonistically.

Video S16. Dynamics of pattern formation in a file of cells, with PIN polarized by auxin efflux and influx acting synergistically.

Supplementary Data

## Abbreviations:

IAA: indole-3-acetic acid
NAA: naphthaleneacetic acid
NOA: naphthoxyacetic acid.
