# Supplementary material for: Auxin-driven patterning with unidirectional fluxes
Source: J Exp Bot. 2015 Jun 27;66(16):5083–102. doi: 10.1093/jxb/erv262 (PMC4513925; doi:10.1093/jxb/erv262)
Supplement: Supplementary Data [file supp_66_16_5083__index.html]

Auxin-driven patterning with unidirectional fluxes — Auxin-driven patterning with unidirectional fluxes — Supplementary Data 

# Auxin-driven patterning with unidirectional fluxes

## Supplementary Data

Data files

- erv262\_SupplementaryText.pdf - Supplementary text, tables and figures
- jexbot146399\_file006.mp4 - Video S01
- jexbot146399\_file007.mp4 - Video S02
- jexbot146399\_file008.mp4 - Video S03
- jexbot146399\_file009.mp4 - Video S04
- jexbot146399\_file010.mp4 - Video S05
- jexbot146399\_file011.mp4 - Video S06
- jexbot146399\_file012.mp4 - Video S07
- jexbot146399\_file013.mp4 - Video S08
- jexbot146399\_file014.mp4 - Video S09
- jexbot146399\_file015.mp4 - Video S10
- jexbot146399\_file016.mp4 - Video S11
- jexbot146399\_file017.mp4 - Video S12
- jexbot146399\_file018.mp4 - Video S13
- jexbot146399\_file019.mp4 - Video S14
- jexbot146399\_file020.mp4 - Video S15
- jexbot146399\_file021.mp4 - Video S16
