# Supplementary material for: Auxin-driven patterning with unidirectional fluxes
Source: J Exp Bot. 2015 Jun 27;66(16):5083–102. doi: 10.1093/jxb/erv262 (PMC4513925; doi:10.1093/jxb/erv262)
Supplement: Supplementary Data [file supp_erv262_erv262_SupplementaryText.pdf]

Auxin-driven Patterning with Unidirectional Fluxes: Supplementary Materials

Mikolaj Cieslak, Adam Runions, and Przemyslaw Prusinkiewicz

Department of Computer Science, University of Calgary
2500 University Dr. N.W., Calgary, AB T2N 1N4 Canada

Contents

S1 Parameters used in simulations **2**

S2 Supplementary figures **4**

S3 Supplementary text **6**

S3.1 Equations describing the network from Figure 6a 6
S3.2 Equations describing steady state of the network from Figure 6c 6
S3.3 Model used to generate Figure 15 7
S3.4 Analysis of pattern emergence in a file of cells with antagonistic or synergistic polarization mechanisms 8
S3.4.1 Antagonistic polarization 9
S3.4.2 Synergistic polarization 11

S4 List of animations **14**

S1 Parameters used in simulations

Parameter	Symbol													
Petri net figure		4b	6a	6b	6c	6c	6c	9	9	9	11a	11a	11b	11b
Simulation figure		5	7a	7b	8a	8b	8c	10a	10b	10c	12a	12b	13a	13b
Video		S2			S5	S6	S7	S8	S9	S10	S11	S12	S13	S14
Parameter	Symbol	Value												
Cell width		···	···	···	···	···	···	1	···	···	···	···	···	···
Cell height		···	···	···	···	···	···	1	···	···	···	···	···	···
Extracell. space width		···	···	···	···	···	···	0.1	···	···	···	···	···	···
Extracell. auxin diffusion		···	···	0.01	···	···	0.1	···	···	···	0.01	···	···	···
Initial auxin concentration		···	···	···	···	···	···	0	···	···	···	···	···	···
AUX/LAX concentration	[AUX]	···	···	···	···	···	···	1	···	···	···	···	···	···
PIN concentration														
— total	$[PIN_{total}]$		···	···	···	···	···	5	···	···	···	···	···	···
— initial/min. in membrane		0	0	0	0.02	0.02	0.02	0.01	0.01	0.01	0.01	0.01	0.01	0.01
Influx rate constant	T_{in}	1	0.1	0.1	1	1	1							
Efflux rate constant	T_{out}	1	0.1	0.1	1	1	1							
Formation of AAUX	T_{in1}							···	···	···	1	···	···	···
Formation of APIN	T_{out1}							···	···	···	1	···	···	···
Influx by AAUX	T_{in2}							···	···	···	1	···	···	···
Efflux by APIN	T_{out2}							2	2	2	1	1	1	1
Auxin production	σ_a	0	0	0.5	0	5	0.00125	0	0.5	0.005	0	0.5	0	0.5
— in L1		15			0	5	1	0	0.5	0.5	0	0.5	0	0.5
— in source		65	1	0.55	10	5	1	0.5	0.5	0.5	2	0.5	2	0.5
Auxin turnover	μ_a	0	0.001	0.1	0.001	1	0.001	0.001	0.1	0.001	0.001	0.1	0.001	0.1
— in sink		1	0.5	0.1	10	1	10	0.1	0.1	0.1	1	0.1	1	0.1
PIN exocytosis														
— constitutive	σ_p	0.005	0.01	0.01	0	0	0	0.05	0.05	0.05	0	0	0.5	0.5
— by tally X	σ_{px}	10^{-5}	0.15	0.15	1	1	1							
— by tally Y	σ_{py}			0.015	0.13	0.13	0.05							
— by APIN	σ_{apin}							4	4	4				
— by AAUX	σ_{aaux}							0.05	0.05	0.05				
— mediated by Z	σ_{pz}										0.5	0.5		
PIN endocytosis														
— constitutive	μ_p	0.05	1	1	0.5	0.5	0.2	2	2	2	2	2		
— mediated by Z	μ_{pz}												2	2
X turnover	μ_x	1	0.1	0.1	1	1	1							
Y turnover	μ_y			0.1	1	1	1							
X-Y annihilation	ν_{xy}	0.1	0.001		250	0.01	ⓐ							
APIN break-up by AAUX	ν_{apin}							4	0.28	ⓑ				
Threshold auxin conc.	a_{th}						15			1.5				
Z production														
— constitutive	σ_z										0	0	1	1
— by APIN	k_1										2	2	0	0
— by AAUX	k_3										0.01	0.01	0.5	0.01
Z turnover														
— constitutive	μ_z										1	1	0.15	0.15
— by APIN	k_2										0	0	2	2
— by AAUX	k_4										0.5	0.01	0.01	0.01
Simulation time step		···	···	···	···	···	···	0.01	···	···	···	···	···	···

ⓐ 0.01 for [A] $\leq a_{th}$, 15 for [A] $> a_{th}$ ⓑ 0.055 for [A] $\leq a_{th}$, 15 for [A] $> a_{th}$

Table S1: Parameter values used in simulations illustrated in the main text. Dotted lines across columns indicate that the same value was used across a range of different models. Blank entries indicate parameters that are not used in specific models.

	Petri net figure	4a	S2	6a	6b
	Simulation figure	S1	S3	S4	S5
	Video	S1	S3	S4	
Parameter	Symbol	Value			
Cell width		1			
Cell height		1			
Extracell. space width			0.1	0.1	0.1
Extracell. auxin diffusion			0.01	0.01	0.01
Initial auxin concentration		0			
AUX/LAX concentration	[AUX]		1	1	1
PIN concentration					
— total	[PIN$_{total}$]			5	5
— initial/min. in membrane		0	0	0	0.05
Influx rate constant	T_{in}	1	1	1	ⓐ
Efflux rate constant	T_{out}	1	1	1	ⓐ
Auxin production	σ_a	0	0	0	0.5
— in L1		15	15	0	
— in source		65	65	5	0.4
Auxin turnover	μ_a	0	0	0.001	0.1
— in sink		1	1	1	0.1
PIN exocytosis					
— constitutive	σ_p	0.005	0.005	0.001	0
— by tally X	σ_{px}	10^{-5}	10^{-6}	0.5	1.5
— by tally Y	σ_{py}				0.375
PIN endocytosis					
— constitutive	μ_p	0.05	0.02	0.1	1
X turnover	μ_x	1	1	1	1
X turnover	μ_y		1		1
X-Y annihilation	ν_{xy}	0.1	0.001	1	

ⓐ The values used in consecutive simulations are:
0.25, 0.5, 1, 10, 150, and 550.

Table S2: Parameter values used in supplementary simulations.

S2 Supplementary figures

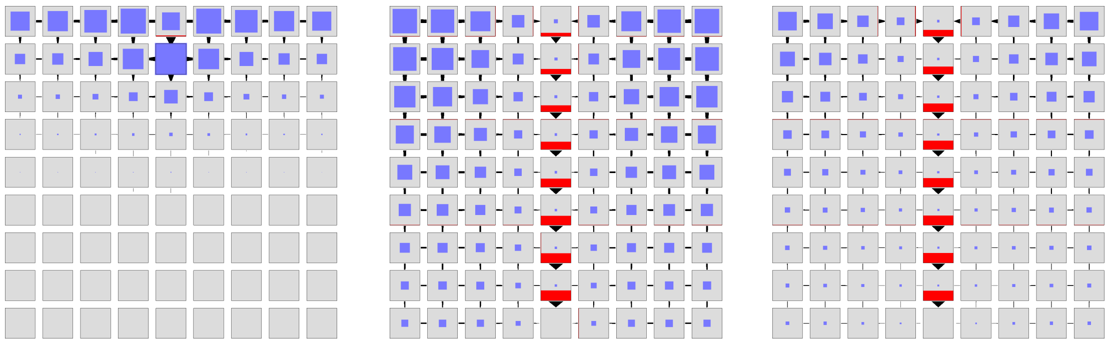

Figure S1: Simulation of canalization using Mitchison's model implemented with tally molecules. The model is defined by the Petri net in Fig. 4a. The cells in the top row are sources of auxin. The centre cell produces auxin at a relatively higher rate. The cell in the centre of the bottom row is an auxin sink.

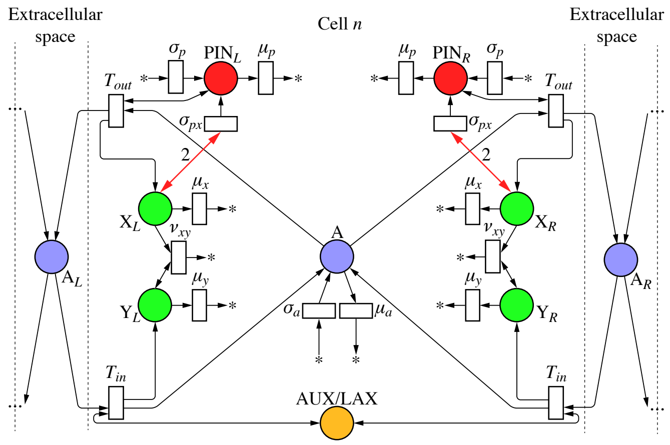

Figure S2: A variant of Mitchison's model with tally molecules and extracellular space, in which PIN allocation is controlled by the ratio of unidirectional fluxes. The network is obtained by replacing the net-flux measuring circuit in Fig. 4b with the flux-ratio measuring circuit from Fig. 2g.

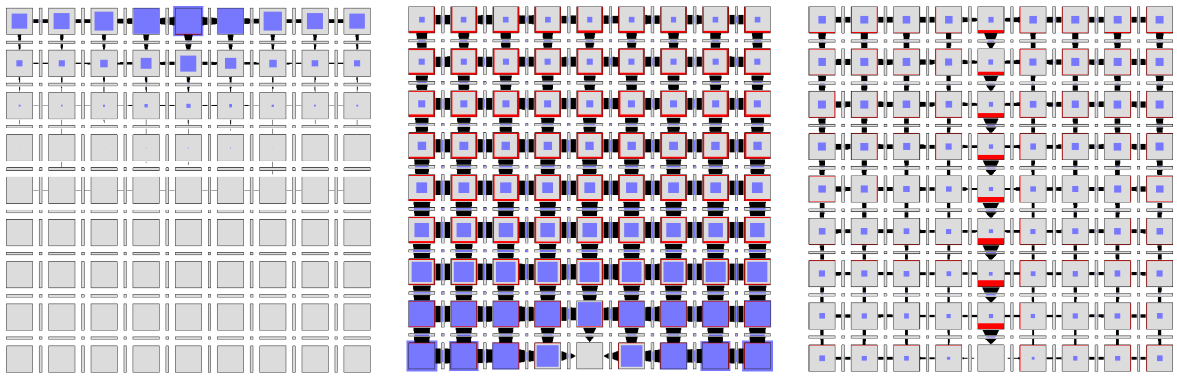

Figure S3: Simulation of canalization using the variant of Mitchison's model defined by the Petri net in Fig. S2. The cells in the top row are sources of auxin. The centre cell produces auxin at a relatively higher rate. The cell in the centre of the bottom row is an auxin sink.

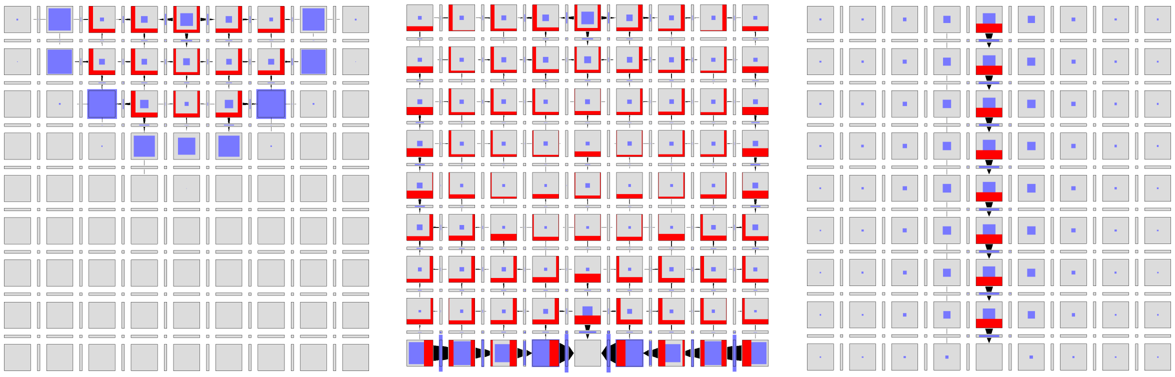

Figure S4: Simulation of canalization with exocytosis controlled by tally molecules. The model is defined by the Petri net in Fig. 6a. The cell in the centre of the top row is an auxin source. The cell in the centre of the bottom row is an auxin sink.

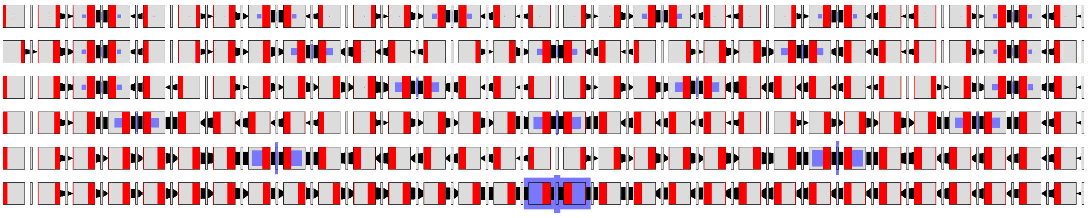

Figure S5: Simulations of convergence point formation with exocytosis controlled by tally molecules. The model is defined by the Petri net in Fig. 6b. The simulations demonstrate the effect of transport efficiency on convergence point spacing. The values of parameters T_{out} and T_{in} increase from top to bottom, causing a decrease in the number of emerging convergence points.

S3 Supplementary text

S3.1 Equations describing the network from Figure 6a

Differential equations for our models are derived directly from their Petri net representation. We provide the equations for the model shown in Fig. 6a as an example.

The change in the concentration of intracellular auxin, [A], is

$$\frac{d[\mathrm{A}]}{dt} = -T_{out}[\mathrm{PIN}_L][\mathrm{A}] - T_{out}[\mathrm{PIN}_R][\mathrm{A}] + T_{in}[\mathrm{AUX}][\mathrm{A}_L] + T_{in}[\mathrm{AUX}][\mathrm{A}_R] + \sigma_a - \mu_a[\mathrm{A}], \tag{S1}$$

where T_{out} is the efflux rate coefficient, and $[\mathrm{PIN}_L]$ and $[\mathrm{PIN}_R]$ are the concentrations of PIN in the left and right membrane segments, respectively. T_{in} is the influx rate coefficient, and [AUX] is the concentration of membrane-localized AUX/LAX in the cell, assumed to be constant. $[\mathrm{A}_L]$ and $[\mathrm{A}_R]$ are extracellular auxin concentrations on the left and right sides of the cell. Finally, σ_a is the auxin production rate, and μ_a is the degradation rate.

We focus on the left membrane segment, as the equations for the right segment are analogous. The change in auxin concentration in the extracellular compartment on the left side of the cell, $[\mathrm{A}_L]$, is

$$\frac{d[\mathrm{A}_L]}{dt} = T_{out}[\mathrm{PIN}_L][\mathrm{A}] - T_{in}[\mathrm{AUX}][\mathrm{A}_L]. \tag{S2}$$

The change in PIN concentration in the left membrane is

$$\frac{d[\mathrm{PIN}_L]}{dt} = \sigma_{px}[\mathrm{X}_L]^2[\mathrm{PIN}] + \sigma_p[\mathrm{PIN}] - \mu_p[\mathrm{PIN}_L], \tag{S3}$$

where σ_{px} is the rate of PIN exocytosis dependent on tally molecule X_L, [PIN] is the concentration of PIN in the cell interior, σ_p is the constitutive rate of exocytosis, and μ_p is the rate of endocytosis. We assume that the total concentration of PIN in the cell, $[\mathrm{PIN}_{total}]$, is constant, so that

$$[\mathrm{PIN}] = [\mathrm{PIN}_{total}] - [\mathrm{PIN}_L] - [\mathrm{PIN}_R]. \tag{S4}$$

Finally, changes in the concentrations of tally molecules are described by the equations

$$\frac{d[\mathrm{X}_L]}{dt} = T_{out}[\mathrm{PIN}_L][\mathrm{A}] - \mu_x[\mathrm{X}_L] - \nu_{xy}[\mathrm{X}_L][\mathrm{Y}_L] \tag{S5}$$

$$\frac{d[\mathrm{Y}_L]}{dt} = T_{in}[\mathrm{AUX}][\mathrm{A}_L] - \nu_{xy}[\mathrm{X}_L][\mathrm{Y}_L], \tag{S6}$$

where μ_x is the degradation rate of X, and ν_{xy} is the rate with which X and Y annihilate each other.

S3.2 Equations describing steady state of the network from Figure 6c

In Section 'Dual polarization' (main text) we noted that that the steady-state concentration of tally molecules in the network from Fig. 6c is described by a quadratic equation. Here we derive this equation and its solutions. As in the main text, we focus on the fluxes through the membrane segment on the left side of the cell.

The steady state concentrations of the tally molecules $[\mathrm{X}_L]$ and $[\mathrm{Y}_L]$ satisfy the equations

$$J_{I\to L} - \nu_{xy}[\mathrm{X}_L][\mathrm{Y}_L] - \mu_x[\mathrm{X}_L] = 0, \tag{S7}$$

$$J_{L\to I} - \nu_{xy}[\mathrm{X}_L][\mathrm{Y}_L] - \mu_y[\mathrm{Y}_L] = 0, \tag{S8}$$

where $J_{I\to L}$ denotes auxin efflux from the cell interior I to the extracellular space L, and $J_{L\to I}$ denotes auxin influx from L to I. If $\nu_{xy} = 0$, these equations have solutions:

$$[\mathrm{X}_L] = \frac{J_{I\to L}}{\mu_x}, \tag{S9}$$

$$[\mathrm{Y}_L] = \frac{J_{L\to I}}{\mu_y}. \tag{S10}$$

In this case, the tally molecules measure directly the efflux and influx of auxin, which reduces the network from Fig. 6c to that from Fig. 6b. To find concentration $[\mathrm{X}_L]$ in the general case $\nu_{xy} \neq 0$, we first calculate $[\mathrm{Y}_L]$ as a function of $[\mathrm{X}_L]$ from Equation S8:

$$[\mathrm{Y}_L] = \frac{J_{L\to I}}{\nu_{xy}[\mathrm{X}_L] + \mu_y}. \tag{S11}$$

After substituting this value into Equation S7, we obtain

$$\mu_x\nu_{xy}[\mathrm{X}_L]^2 + (\mu_x\mu_y - \nu_{xy}\phi_{I\to L})[\mathrm{X}_L] - \mu_y J_{I\to L} = 0, \tag{S12}$$

where $\phi_{I\to L} = J_{I\to L} - J_{L\to I}$. This quadratic equation has positive solution

$$[\mathrm{X}_L] = \frac{(\nu_{xy}\phi_{I\to L} - \mu_x\mu_y) + \sqrt{(\nu_{xy}\phi_{I\to L} - \mu_x\mu_y)^2 + 4\mu_x\mu_y\nu_{xy}J_{I\to L}}}{2\mu_x\nu_{xy}}. \tag{S13}$$

Likewise, $[\mathrm{Y}_L]$ satisfies the equation

$$\mu_y\nu_{xy}[\mathrm{Y}_L]^2 + (\mu_x\mu_y + \nu_{xy}\phi_{I\to L})[\mathrm{Y}_L] - \mu_x J_{L\to I} = 0, \tag{S14}$$

which has positive solution

$$[\mathrm{Y}_L] = \frac{-(\nu_{xy}\phi_{I\to L} + \mu_x\mu_y) + \sqrt{(\nu_{xy}\phi_{I\to L} + \mu_x\mu_y)^2 + 4\mu_x\mu_y\nu_{xy}J_{L\to I}}}{2\mu_y\nu_{xy}}. \tag{S15}$$

Unfortunately, Equations S13 and S15 are difficult to interpret due to their relatively complex form. For this reason, we have not analyzed the network from Fig. 6c directly, but exploited its similarities to the networks from Fig. 6a and 6b instead.

S3.3 Model used to generate Figure 15

In Section 'Discussion' (main text) we observed that the key modes of auxin-driven patterning can be distinguished by contrasting roles of the auxin influx, which may act either antagonistically of synergistically with the efflux. These possibilities are illustrated in Fig. 15. To create it, we developed a simplified model of cell polarization, in which PIN allocation to the membranes is controlled directly by unidirectional fluxes. Here we present the equations defining this model (due to the simplification, it cannot be specified by a Petri net).

We consider a single cell sandwiched between two extracellular compartments, top (T) and bottom (B). PIN concentration in the membrane facing either compartment is controlled by a linear combination of auxin efflux and influx. In the case of the top membrane (an analogical formula applies to the bottom membrane), this combination is

$$Q_{I\to E_T} = J_{I\to E_T} + \alpha J_{E_T\to I}. \tag{S16}$$

Auxin efflux $J_{I\to E_T}$ and influx $J_{E_T\to I}$ thus act synergistically if $\alpha > 0$ and antagonistically if $\alpha < 0$. Given $Q_{I\to E_T}$, PIN allocation to the top membrane changes according to the equation

$$\frac{d[\mathrm{PIN}_T]}{dt} = \begin{cases} \sigma_{pq}[\mathrm{PIN}]Q^2_{I\to E_T} + \sigma_p[\mathrm{PIN}] - \mu_p[\mathrm{PIN}_T] & \text{if } Q_{I\to E_T} \geq 0 \\ \sigma_p[\mathrm{PIN}] - \mu_p[\mathrm{PIN}_T] & \text{if } Q_{I\to E_T} < 0, \end{cases} \tag{S17}$$

where [PIN] is the concentration of PIN not allocated to either membrane:

$$[\mathrm{PIN}] = [\mathrm{PIN}_{total}] - [\mathrm{PIN}_T] - [\mathrm{PIN}_B]. \tag{S18}$$

The model is completed by the equations describing unidirectional fluxes. The efflux from the cell to the top extracellular compartment is calculated as

$$J_{I\to E_T} = T_{out}[\mathrm{A}][\mathrm{PIN}_T], \tag{S19}$$

where T_{out} is an efflux rate constant and [A] is auxin concentration in the cell. Changes in this concentration are calculated as the sum of net fluxes between the cell and the extracellular compartments, as well as the production (with rate σ_a) and turnover (with rate μ_a) of auxin:

$$\frac{d[\mathrm{A}]}{dt} = (J_{I\to E_T} - J_{E_T\to I}) + (J_{I\to E_B} - J_{E_B\to I}) + \sigma_a - \mu_a[\mathrm{A}]. \tag{S20}$$

The influx from the top extracellular compartment is calculated as

$$J_{E_T\to I} = T_{in}[\mathrm{A}_T][\mathrm{AUX}], \tag{S21}$$

where T_{in} is an influx rate constant, $[\mathrm{A}_T]$ is auxin concentration in the extracellular compartment, and [AUX] is the AUX/LAX concentration in the cell (assumed to be constant).

The values of model parameters, initial conditions and boundary conditions used to create Fig. 15 are listed in Table S3. The same parameter values, except σ_a and μ_a, were also employed in the simulations shown in Figs. S7 and S9, which are discussed in Section S3.4.

Parameters									Initial conditions				Boundary conditions	
σ_{pq}	σ_p	μ	T_{out}	T_{in}	σ_a	μ_a	[AUX]	$[\mathrm{PIN}_{total}]$	[A]	[PIN]	$[\mathrm{PIN}_T]$	$[\mathrm{PIN}_B]$	$[\mathrm{A}_T]$	$[\mathrm{A}_B]$
0.0001	0.01	0.1	0.5	0.5	0	0	1	5	0	5	0	0	15	10

Table S3: Parameter values, initial conditions and boundary conditions used in the simplified model of cell polarization shown in Fig. 15.

S3.4 Analysis of pattern emergence in a file of cells with antagonistic or synergistic polarization mechanisms

Fig. 15 illustrates the effects of antagonistic vs. synergistic polarization on an isolated cell. To better understand how these polarization modes lead to pattern formation, we considered a linear arrangement of cells with periodic boundary conditions (i.e., the first cell and the last cell are assumed to be neighbours). The individual cells are modelled as described in Section S3.3, using the same parameter values (Table S3), except for $\sigma_a = 10$ and $\mu_a = 0.1$. The visualizations are extended to show the rates of change in PIN concentrations in the left and right membranes ($d[\mathrm{PIN}_{L,R}]/dt$) and the net flux ($\phi_{L,R}$) through these membranes. Fig. S6 explains the visualization convention used.

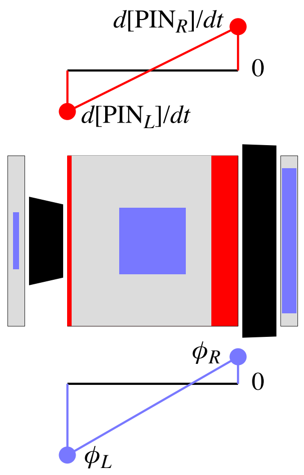

Figure S6: Extended visualization of a cell. Auxin concentrations in the cell (blue square) and in the extracellular space (blue rectangles), concentrations of PIN allocated to the membrane segments (red rectangles), and the efflux and influx of auxin (black trapezoids) are visualized as in Fig. 3b. The red circles and the associated vertical lines indicate the rates of PIN change in the left ($d[\mathrm{PIN}_L]/dt$) and right ($d[\mathrm{PIN}_R]/dt$) membrane segments. The blue circles and vertical lines indicate net auxin fluxes (ϕ_L and ϕ_R) through the respective membranes. In each case, the black horizontal line labeled 0 separates positive and negative values. The slope of lines connecting pairs of circles represents the difference between values at the left and right membrane segments.

S3.4.1 Antagonistic polarization

Figure S7 and Video S15 illustrate pattern formation with the auxin efflux and influx acting on PIN polarization antagonistically. At the beginning of simulation cells are not polarized. The initial auxin concentration in the first cell is positive, and in other cells is equal to zero (Fig. S7a). In the absence of polarization, auxin fluxes have diffusive character, and auxin flows away from the first cell (while also being produced in all cells, Fig. S7b). With the auxin efflux and influx acting antagonistically, PIN in the adjacent cells becomes polarized in the direction of net auxin flow. This interplay between auxin flow and PIN polarization repeats in the subsequent cells, propagating PIN polarization away from the first cell through a "domino effect" (Fig. S7c; note the concurrent slopes of red and blue lines above and below each cell). These propagating polarizations converge at the farthest location from the first cell and produce an auxin maximum there (Fig. S7d). Compared to the initial state, concentration differences are now reversed: the cell with the highest initial concentration has become the cell with the lowest concentration. The pattern in Fig. S7e represents a stable steady-state configuration of the system.

The described process is robust, in the sense that a similar pattern, with a single convergence point at the location opposing the first cell, is produced in shorter or longer files of cells (results not shown). If the initial conditions are changed so that the first cell has a lower auxin concentration than others, the convergence point emerges in the first cell (Fig. S8). In either case, the antagonistic action of auxin efflux and influx results in a coordinated polarization of cells, with PIN oriented towards a single convergence point. This point is located away from the initial auxin maximum, or at the initial auxin minimum.

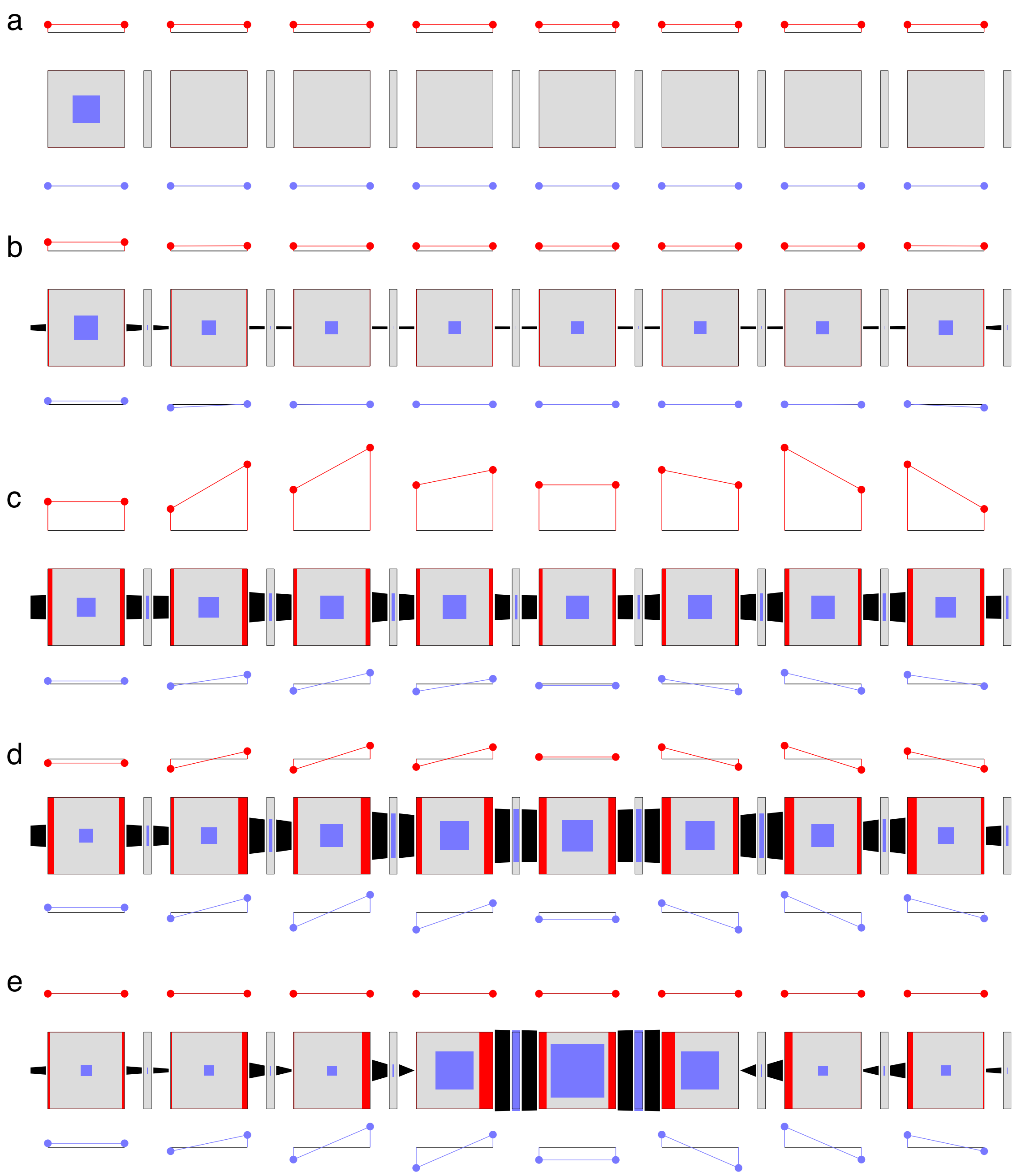

Figure S7: Dynamics of pattern formation in a file of cells with PIN polarized by auxin efflux and influx acting antagonistically ($\alpha = -0.135$ in Equation S16). The first cell has a higher auxin concentration than the remaining cells. The initial state (a), three intermediate states (b–d) and the final steady state (e) of the simulation are shown.

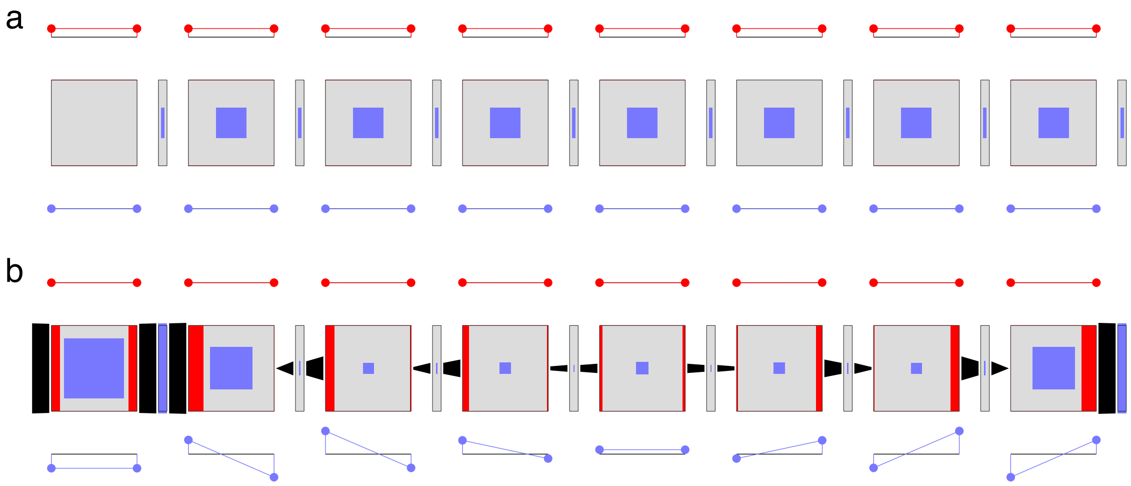

Figure S8: Pattern formation in a file of cells with PIN polarized by auxin efflux and influx acting antagonistically. In contrast to Fig. S7, the first cell has lower initial concentration than the remaining cells. The initial state (a) and the final steady state (b) of the simulation are shown.

S3.4.2 Synergistic polarization

Figure S9 and Video S16 illustrate pattern formation with the auxin efflux and influx acting synergistically. The simulations of antagonistic and synergistic polarization differ only by the value of parameter α, which is now positive ($\alpha = 0.135$). The initial state is the same, with the first cell having higher auxin concentration than other cells (Fig. S9a). As in the previous simulation, the initial fluxes are directed away from the first cell and propagate to the neighbouring cells. A gradient of auxin concentrations and total fluxes results, decreasing away from the first cell (Fig. S9b). Synergistic polarization implies that PIN will be localized preferentially to the membranes with a higher total flux. As the gradient of fluxes coincides with the gradient of auxin concentration, PIN becomes polarized towards the neighbouring cell with a higher auxin concentration (Fig. S9c). This polarization is opposite to the net auxin flux (note the opposite slope of red and blue lines in Fig. S9c). Eventually, the direction of net flux reverses to coincide with polarization (Fig. S9d; note that the slopes of red and blue lines now concur). As a result, a convergence point forms in the first cell, i.e., at the location of the initial auxin maximum (Fig. S9e).

If the first cell has a lower initial auxin concentration than other cells, the convergence point emerges in the cell that is furthest away from the first cell (Fig. S10). Independently of the initial conditions, in longer cell files additional, approximately equally spaced convergence points may emerge, as the depletion of auxin in the proximity of one convergence points can lead to the formation of the next convergence point at some distance away. The number of convergence points increases as the length of the file increases (Fig. S11) or the value of parameter α decreases (result not shown).

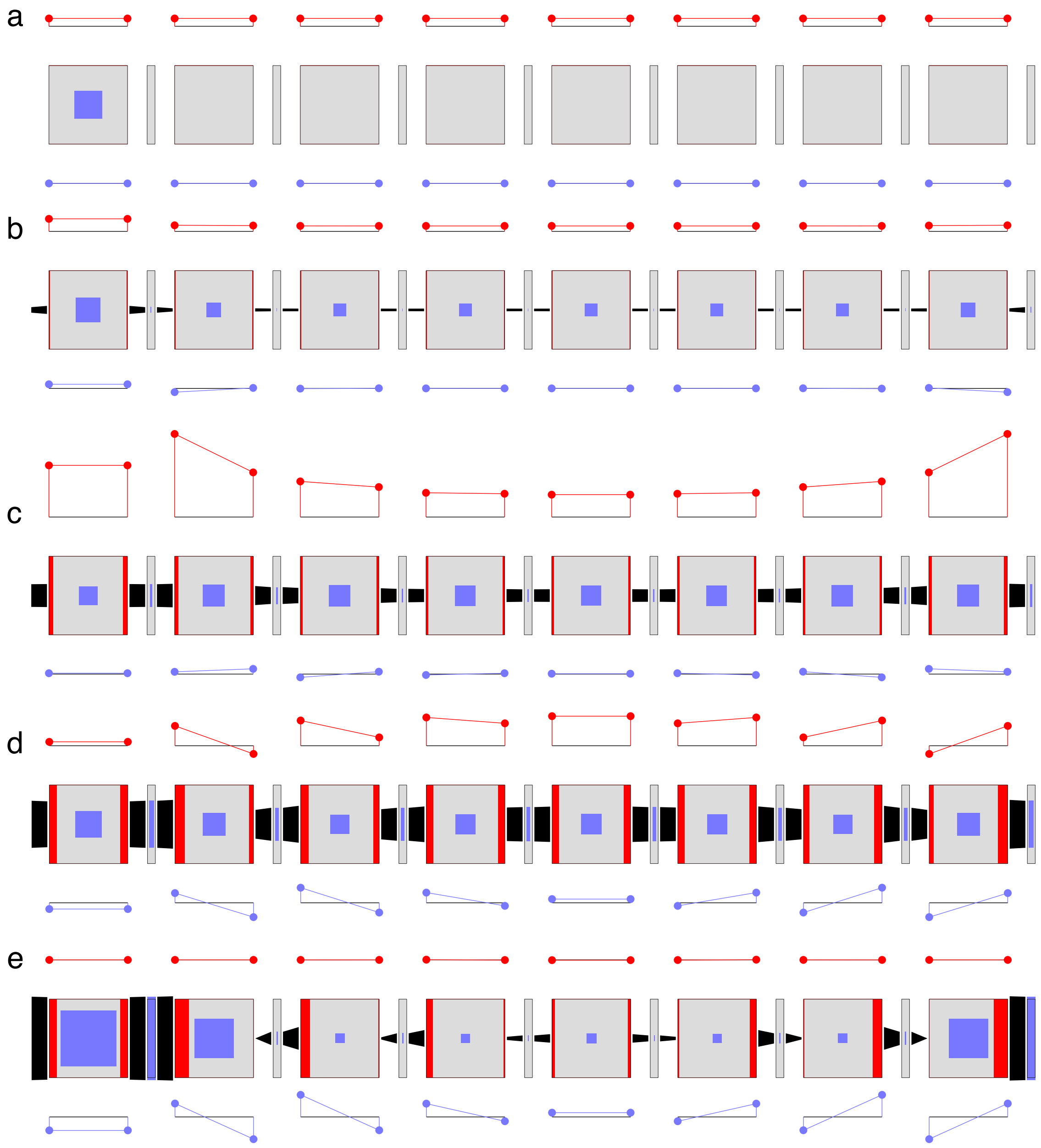

Figure S9: Dynamics of pattern formation in a file of cells with PIN polarized by auxin efflux and influx acting synergistically ($\alpha = 0.135$ in Equation S16). The initial state (a), three intermediate states (b–d) and the final steady state (e) of the simulation are shown.

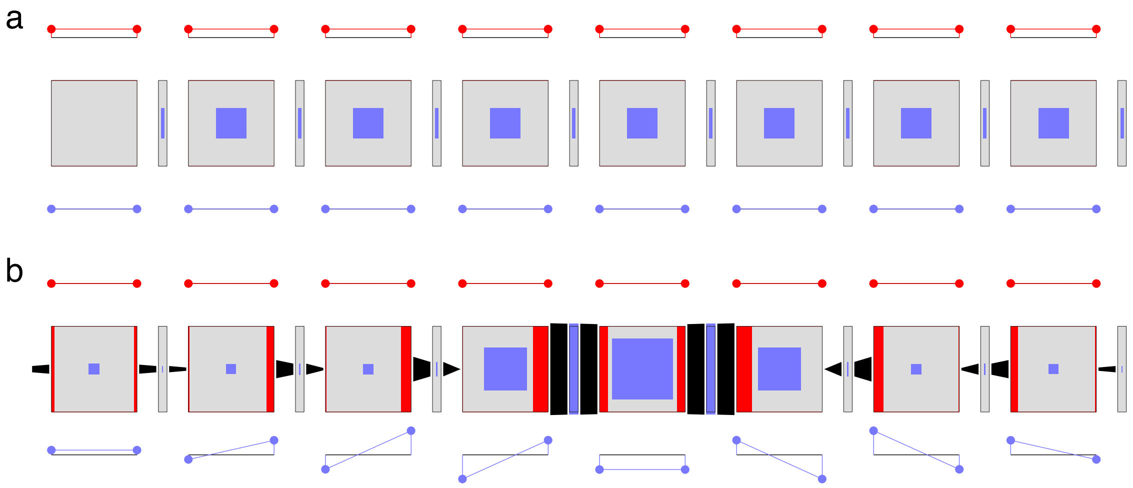

Figure S10: Pattern formation in a file of cells with PIN polarized by auxin efflux and influx acting synergistically. In contrast to Fig. S9, the first cell has lower initial concentration than the remaining cells. The initial state (a) and the final steady state (b) of the simulation are shown.

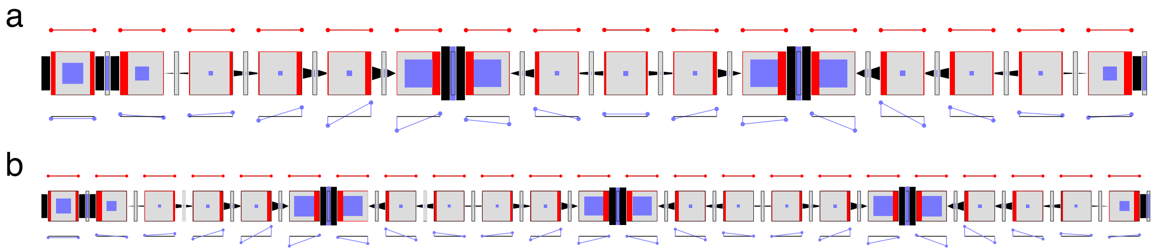

Figure S11: Convergence points produced in cell files of different length. PIN is polarized by auxin efflux and influx acting synergistically. The final steady states of the simulations are shown for (a) 16 cells and (b) 23 cells. The value of $\alpha = 0.5$ was chosen so that the convergence points are approximately equidistant.

S4 List of animations

Video S1: Canalization using Mitchison's model implemented with tally molecules. The model is defined by the Petri net in Fig. 4a. Selected frames from this simulation are shown in Fig. S1.

Video S2: Canalization using Mitchison's model with tally molecules and extracellular space. The model is defined by the Petri net in Fig. 4b. Selected frames from this simulation are shown in Fig. 5.

Video S3: Canalization using a variant of Mitchison's model with tally molecules and extracellular space, in which PIN allocation is controlled by the ratio of unidirectional fluxes instead of the net flux. The model is defined by the Petri net in Fig. S2. Selected frames from this simulation are shown in Fig. S3.

Video S4: Canalization with exocytosis controlled by tally molecules. The model is defined by the Petri net in Fig. 6a. Selected frames from this simulation are shown in Fig. S4.

Video S5: Canalization with exocytosis controlled by tally molecules. The model is defined by the Petri net in Fig. 6c. Selected frames from this simulation are shown in Fig. 8a.

Video S6: Convergence point formation with exocytosis controlled by tally molecules. The model is defined by the Petri net in Fig. 6c. Selected frames from this simulation are shown in Fig. 8b.

Video S7: Dual-polarization with exocytosis controlled by tally molecules. The model is defined by the Petri net in Fig. 6c. Selected frames from this simulation are shown in Fig. 8c.

Video S8: Canalization with exocytosis controlled by influx and efflux carriers bound to auxin in the membrane. The model is defined by the Petri net in Fig. 9. Selected frames from this simulation are shown in Fig. 10a.

Video S9: Convergence point formation with exocytosis controlled by influx and efflux carriers bound to auxin in the membrane. The model is defined by the Petri net in Fig. 9. Selected frames from this simulation are shown in Fig. 10b.

Video S10: Dual polarization with exocytosis controlled by influx and efflux carriers bound to auxin in the membrane. The model is defined by the Petri net in Fig. 9. Selected frames from this simulation are shown in Fig. 10c.

Video S11: Canalization with exocytosis controlled by influx and efflux carriers that act via a mediating molecule. The model is defined by the Petri net in Fig. 11a. Selected frames from this simulation are shown in Fig. 12a.

Video S12: Convergence point formation with exocytosis controlled by influx and efflux carriers that act via a mediating molecule. The model is defined by the Petri net in Fig. 11a. Selected frames from this simulation are shown in Fig. 12b.

Video S13: Canalization with endocytosis controlled by influx and efflux carriers that act via a mediating molecule. The model is defined by the Petri net in Fig. 11b. Selected frames from this simulation are shown in Fig. 13a.

Video S14: Convergence point formation with endocytosis controlled by influx and efflux carriers that act via a mediating molecule. The model is defined by the Petri net in Fig. 11b. Selected frames from this simulation are shown in Fig. 13b.

Video S15: Dynamics of pattern formation in a file of cells, with PIN polarized by auxin efflux and influx acting antagonistically. The model is defined in Section S3.3. Selected frames from this simulation are shown in Fig. S7.

Video S16: Dynamics of pattern formation in a file of cells, with PIN polarized by auxin efflux and influx acting synergistically. The model is defined in Section S3.3. Selected frames from this simulation are shown in Fig. S9.
